# Lobal Burden of LDL‐C‐Related Ischemic Stroke: Unveiling Socioeconomic Reversals, Age‐Sex Disparities, and the Looming Threat of Slowing Cohort Improvements

**DOI:** 10.1002/brb3.71911

**Published:** 2026-11-13

**Authors:** Xiaodong Yu, Yi Zhang, Nannuan Liu, Yao Liu, Genshan Gao

**Affiliations:** ^1^ Department of Neurology Renmin Hospital Hubei University of Medicine Shiyan P. R. China; ^2^ Department of Geriatrics Renmin Hospital Hubei University of Medicine Shiyan P. R. China

**Keywords:** age‐period‐cohort analysis, global burden, health disparities, ischemic stroke, LDL‐C, socioeconomic factors

## Abstract

**Background:**

Ischemic stroke persists as a primary driver of global disease burden and death, wherein heightened levels of low‐density lipoprotein cholesterol (LDL‐C) constitute a critical, alterable risk determinant. Despite advances in lipid‐lowering therapies, substantial inequalities in the burden of LDL‐C‐associated ischemic stroke (HLIS) continue to exist across different geographical areas and socioeconomic strata.

**Methods:**

Drawing upon data from the 2021 Global Burden of Disease Study, we comprehensively assessed the burden of HLIS spanning 1990 through 2021. This evaluation encompassed 204 distinct countries and territories, stratified by biological sex and divided into 25 specific age cohorts. Key metrics analyzed included the age‐standardized mortality rate (ASMR) alongside the disability‐adjusted life year (DALY) rate. Furthermore, to elucidate the underlying drivers of observed temporal trends, we performed decomposition analyses, age–period–cohort modeling, and assessments of health inequity.

**Results:**

From 1990 to 2021, global HLIS‐related deaths increased by 43.03% (654,503 to 936,192), while age‐standardized rates declined annually (ASMR: −2.06%; DALYs: −1.72%). The steepest declines occurred in high‐SDI regions (e.g., Western Europe [ASMR: −4.65%/year]). In contrast, low‐SDI regions, particularly Southern Sub‐Saharan Africa, experienced rising burdens (ASMR: +1.03%/year). A socioeconomic reversal was observed, with burden shifting toward lower‐income populations (Concentration Index changed from 0.08 to −0.09). Population aging and growth drove the absolute rise in cases, especially in low‐resource settings. Sex disparities were notable, with higher mortality in elderly women and greater DALYs in middle‐aged men. Birth cohort analysis suggested slowing improvements among younger generations.

**Conclusions:**

Although age‐standardized HLIS burden has declined globally, persistent inequalities across regions, sexes, and socioeconomic groups reflect ongoing disparities in healthcare access and lipid management. Targeted interventions addressing demographic shifts and strengthening primary prevention in resource‐limited settings are urgently needed to reduce future disease burden.

## Graphical abstract

Using Global Burden of Disease 2021 data from 204 countries and territories, we assessed the global burden of high LDL‐Crelated ischemic stroke from 1990 to 2021. Although age‐standardized mortality and DALY rates declined, absolute deaths and DALYs increased by 43.03% and 44.55%, respectively, primarily driven by population growth and aging. The burden shifted toward lower‐SDI populations, with marked agesex disparities and slowing improvements among recent birth cohorts, underscoring the need for equitable lipid management and demographically tailored primary prevention.

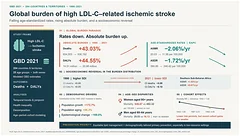

## 1 Introduction

Ischemic stroke continues to represent a primary driver of worldwide morbidity and death, posing a substantial and growing burden on public health systems worldwide (Zhao et al. 2025; Hua et al. 2025; Feigin et al. 2024). Although advances in acute treatment and secondary prevention have been achieved, significant geographical disparities persist in both the incidence and overall burden of this disease (An et al. 2025; Kisa and Kisa 2021). While high‐income countries have reported declining age‐standardized mortality rates (ASMR), largely attributed to optimized healthcare resource allocation and comprehensive risk factor management. Numerous low‐ and middle‐income areas persist in facing a growing strain, driven by constrained healthcare systems and inadequate management of risk determinants (Hasani et al. 2023; Yan et al. 2016). Furthermore, global population aging is contributing to a rising absolute number of ischemic stroke cases (Chen et al. 2025), underscoring the urgent need for a detailed epidemiological analysis to inform targeted prevention strategies.

Elevated low‐density lipoprotein cholesterol (LDL‐C) is a well‐established, modifiable risk factor for atherosclerotic cardiovascular disease and is closely linked to the pathogenesis of ischemic stroke.

High levels of LDL‐C represent a confirmed and adjustable risk element for atherosclerotic cardiovascular conditions, showing a strong association with the development mechanisms of ischemic stroke (Bubb et al. 2022; Chen et al. 2024). Mechanistically, LDL‐C contributes to endothelial impairment, atherosclerotic lesions, and thrombosis, thereby elevating the risk of cerebral infarction (Wulff et al. 2023). Despite the proven efficacy of lipid‐lowering therapies such as statins in reducing LDL‐C levels and subsequent stroke incidence (Wang et al. 2024), global treatment coverage and achievement of target levels remain inadequate, particularly in resource‐limited settings (Annemans et al. 2020). Consequently, a systematic evaluation of the disease burden specifically attributable to LDL‐C‐related ischemic stroke (HLIS) is crucial for guiding clinical practice and shaping effective public health policies.

The Global Burden of Disease (GBD) initiative delivers extensive epidemiological insights regarding stroke, however, significant knowledge gaps remain regarding the HLIS‐specific burden. First, prior analyses have often focused on the overall stroke burden, lacking an in‐depth exploration of socioeconomic determinants, age‐sex disparities, and birth cohort effects specific to HLIS (Kisa and Kisa 2021). Second, although the epidemiological transition has shifted the stroke burden toward low‐ as well as middle‐income countries (Li et al. 2024), the underlying drivers of this trend for HLIS are not fully elucidated. Additionally, the rising prevalence of dyslipidemia among younger populations (Kaneko et al. 2021) suggests a potential shift in future disease patterns, yet long‐term risk projections based on birth cohort analyses are limited. Such deficiencies impede the formulation of interventions meticulously customized for heterogeneous at‐risk cohorts.

Drawing upon the GBD 2021 dataset, this investigation conducts a comprehensive evaluation of the worldwide impact attributable to HLIS, encompassing 204 distinct nations and territories over the three‐decade span from 1990 through 2021. By integrating health inequality metrics, age‐period‐cohort modeling, and decomposition analysis, we aim to: (1) elucidate the spatiotemporal evolution of the HLIS burden in relation to socioeconomic development; (2) quantify age‐ and sex‐specific disparities in its distribution; and (3) assess risk trends across successive birth cohorts to identify emerging high‐risk populations. The findings are intended to provide critical evidence for optimizing global lipid management strategies and reducing related health inequities, thereby contributing to more equitable advancements in stroke prevention.

## 2 Methods

### 2.1 Data Sources

This secondary analysis leveraged the most recent publicly available data from the GBD, Injuries, and Risk Factors Study (GBD) 2021. GBD 2021 delivers exhaustive mortality estimates covering 288 distinct causes across 204 nations and territories. These data are stratified by 25 age cohorts (ranging from birth to ≥95 years), sex (male, female, and both sexes combined), and year (1990–2021) (Wu et al. 2024; CJL 2024). Furthermore, the included nations and territories are organized into 21 geographic regions and seven overarching super‐regions.

The GBD initiative utilizes a unified meta‐regression modeling system, specifically DisMod‐MR 2.1, to guarantee internal coherence while mitigating data heterogeneity and filling informational voids by integrating evidence from 100,983 unique sources (GBD 2017 Disease and Injury Incidence and Prevalence Collaborators 2018). This framework generates estimates for incidence, prevalence, mortality, years of life lost (YLL), years lived with disability (YLD), as well as disability‐adjusted life years (DALYs) for 371 diseases and injuries. As the aggregate of YLL and YLD, DALYs serve as the primary metric for total disease burden. YLL figures are computed by multiplying cause‐specific death counts, stratified by age, sex, geography, and year, against standard life expectancy values at the age of demise. Conversely, YLD estimates result from multiplying the prevalence of specific disease sequelae by their assigned disability weights. Comprehensive descriptions of these analytical procedures are available in earlier GBD methodological reports (Abbas et al. 2020).

In GBD 2021, a “risk factor” is defined as any attribute causally associated with increased disease incidence or prevalence. This investigation evaluated a total of 88 distinct risk factors and clustered risks encompassing behavioral, environmental/occupational, and metabolic domains (Aboagye et al. 2024). High LDL‐C is classified as a key metabolic risk factor. The exposure threshold for high LDL‐C was defined as ≥2.6–3.0 mmol/L, consistent with the GBD 2021 definition for this modifiable physiological abnormality. Additional established metabolic risk factors encompass increased systolic blood pressure, elevated fasting plasma glucose levels, higher body mass index, and compromised renal function.

To stratify the study population, we employed the Socio‐demographic Index (SDI), a composite metric reflecting regional socioeconomic progression. This index is derived from three core parameters: lagged distributed income per capita, mean years of education for individuals aged 15 and older, and the total fertility rate among women under 25. For analytical purposes, the 21 GBD regions were classified into five distinct SDI quintiles: high (>0.81), high‐middle (0.70–0.81), middle (0.61–0.69), low‐middle (0.46–0.60), and low (<0.46) (Collaborators CACD 2023).

All estimates from GBD 2021 are publicly accessible via the Global Health Data Exchange (GHDx) (https://vizhub.healthdata.org/gbd‐results/). Given that this research constitutes a secondary analysis of de‐identified, aggregated datasets, it was exempt from further ethical scrutiny. The original GBD study secured ethical clearance from the University of Washington's Institutional Review Board, which included a waiver for informed consent requirements (Roth et al. 2020).

### 2.2 Definition of HLIS

In the GBD comparative risk assessment framework, HLIS (high LDL‐C‐related ischemic stroke) refers to the subset of ischemic stroke burden that is causally attributable to elevated LDL‐C exposure. Elevated LDL‐C is classified as a Level 2 metabolic risk factor within the GBD risk factor hierarchy, with exposure defined as LDL‐C levels exceeding the TMREL of 0.7–1.3 mmol/L (approximately 27–50 mg/dL) (Aboagye et al. 2024). Ischemic stroke is defined as a Level 4 cause in the GBD cause hierarchy, characterized by cerebral infarction resulting from thrombosis or embolism. The causal association between high LDL‐C and ischemic stroke is supported by convincing evidence from epidemiological studies and randomized controlled trials, and is incorporated into GBD's risk‐outcome pairs based on established criteria (Feigin et al. 2024). Within this framework, the PAF for ischemic stroke due to increased LDL‐C is estimated by integrating exposure prevalence with relative risk estimates from pooled cohort studies and meta‐analyses. The number of HLIS‐related deaths and DALYs is then calculated as the product of total ischemic stroke burden and the corresponding PAF (GBD 2017 Disease and Injury Incidence and Prevalence Collaborators 2018). Mechanistically, elevated LDL‐C contributes to endothelial dysfunction, atherosclerotic plaque formation, and thrombosis via qualitative alterations of lipoproteins, including oxidized LDL, small dense LDL, and electronegative LDL, which promote subendothelial cholesterol accumulation and inflammatory processes characteristic of atherosclerotic lesions (Benitez et al. 2024).

### 2.3 Health Inequality Assessment

To quantify socioeconomic inequality in HLIS burden, we employed the Slope Index of Inequality (SII) and the Concentration Index (CI) from 1990 to 2021. The SII measures absolute inequality, quantifying the disparity in health status between populations at the highest and lowest ends of the socioeconomic spectrum, weighted across the entire population distribution (Abu‐Gharbieh et al. 2024). The CI quantifies relative inequality by assessing the extent to which health outcomes are disproportionately distributed across socioeconomically disadvantaged versus advantaged populations. Specifically, the 204 countries and territories were ranked by SDI from the most disadvantaged (Rank 0) to the most advantaged (Rank 1), and each country was assigned a population‐weighted relative rank (ridit score) defined as the midpoint of its cumulative share of the total population in the SDI‐ranked distribution, so that the ranking accounts for differences in population size. The SII was calculated by regressing the age‐standardized HLIS rates of all countries on these relative rank scores using a weighted linear regression model, with the slope of the regression line representing the absolute difference in predicted burden between the theoretically most advantaged and most disadvantaged populations (Organization WH 2013). The CI was derived from the concentration curve, which plots the cumulative proportion of HLIS deaths or DALYs against the cumulative proportion of the population ranked by SDI, and was computed as twice the area between the concentration curve and the line of equality—equivalently, from the covariance between the burden measure and the fractional SDI rank—yielding a relative measure ranging from −1 to +1 (Organization WH 2013). For interpretation, positive SII or CI values indicate that the HLIS burden is concentrated among populations with higher SDI, whereas negative values indicate that the burden is disproportionately concentrated among lower‐SDI, more socioeconomically disadvantaged populations. All inequality analyses were performed in R (version 4.4.2) using the JD_GBDR package (V2.37; JingDing Medical Technology Co., Ltd), which implements the population‐weighted ranking, regression, and covariance‐based procedures described above. Furthermore, frontier analysis was employed to determine the maximum attainable health performance benchmarked against a specific location's SDI (Yu et al. 2024).

### 2.4 Age‐Period‐Cohort (APC) Modeling

APC analysis was conducted to disentangle temporal trends into three dimensions: age effects (changes in risk across the lifespan), period effects (impacts of external factors like medical advancements), and cohort effects (the accumulated risk experienced by specific birth cohorts) (Huang et al. 2024). Based on GBD 2021 estimates, participants were stratified into sequential age cohorts., calendar periods, and overlapping birth cohorts. The intrinsic estimator method was used to resolve model identification issues. To evaluate the statistical significance of both the net drift (representing the overall annual percent change) and local drifts (denoting age‐specific annual percent changes), the Wald χ^2^ test was employed. Relative risk ratios were calculated for period and cohort effects relative to reference groups (Fan et al. 2023).

### 2.5 Joinpoint Regression Analysis

Trends in HLIS burden over time were analyzed using joinpoint regression (Joinpoint software version 4.9.1.0, National Cancer Institute). This method identifies points where a statistically significant change in the linear trend of age‐standardized rates occurs, fitting the simplest model necessary to describe the data. A maximum of five joinpoints were permitted over the 1990–2021 study period, with at least two observations required between adjacent joinpoints and at both ends of the series. The optimal number and location of joinpoints were determined using the permutation test procedure implemented in the software, which sequentially compares models with an increasing number of joinpoints against the simpler model at an overall significance level of α = 0.05, with the Bonferroni correction applied to control the family‐wise error rate (Kim et al. 2000). For each segment between consecutive joinpoints, the annual percent change (APC) was estimated from the slope of a log‐linear regression of the age‐standardized rate on calendar year. The average annual percent change (AAPC) served as a summary metric to characterize the overall trend across the full study duration from 1990 to 2021; it was computed as a geometrically weighted average of the segment‐specific APCs, with weights proportional to the length (in years) spanned by each segment, and the corresponding 95% confidence intervals were derived from the standard error of the weighted regression slope using the parametric method (Clegg et al. 2009). Statistical significance was determined by evaluating whether the estimated AAPC deviated significantly from zero (*p* < 0.05).

### 2.6 Statistical Analysis Framework

Regional and Socio‐demographic Index (SDI) stratifications were applied to compute ASMR and age‐standardized disability‐adjusted life year rates (ASDR) attributable to LDL‐C‐related ischemic stroke (HLIS). To characterize temporal dynamics, we modeled the natural logarithm of these age‐standardized metrics against calendar years via linear regression: ln(rate) = α + β × year + ε, where β denotes the slope parameter. The estimated annual percentage change (EAPC) was subsequently derived as 100 × [exp(β) – 1], with corresponding 95% confidence intervals (CI) extracted directly from the regression output. Statistical significance for trend direction was defined by EAPC estimates whose 95% CIs excluded zero: values above zero signified a significant increase, whereas those below zero indicated a significant decline. Furthermore, Spearman's rank correlation analysis quantified the relationship between SDI levels and HLIS‐associated ASMR and ASDR burdens. All statistical computations were executed in R (version 4.4.2), utilizing the JD_GBDR package (V2.37; JingDing Medical Technology Co., Ltd).

## 3 Results

### 3.1 Global and Regional Trends

In 2021, HLIS accounted for 936,192 fatalities worldwide (95% UI: 299,535–1,614,294), representing a 43.03% increase compared to 1990 (654,503 deaths) (Table 1). Regionally, East Asia (307,724 deaths, 95% UI: 95,030–538,672) and Eastern Europe (101,522 deaths, 95% UI: 34,052–169,992) had the highest death counts, likely due to aging populations and suboptimal hypertension management, whereas Oceania (497 deaths), Andean Latin America (2538 deaths), and Australasia (2572 deaths) had the lowest burdens. The global ASMR was 11.38 per 100,000 population (95% UI: 3.62–19.65), with Eastern Europe (28.15 per 100,000), North Africa, Middle East (19.78 per 100,000), and Central Asia (18.33 per 100,000) ranking highest. The lowest ASMRs were observed in high‐income regions, specifically Australasia (3.9 per 100,000) and North America (4.0 per 100,000) (Table 1, Figure 1A), reflecting disparities in healthcare resources and preventive measures. From 1990 to 2021, the EAPC in ASMR was ‐2.06% (95% UI: −2.15 to −1.96), with the steepest declines observed in high‐income Asia Pacific (−4.82%) as well as Western Europe (−4.65%). In contrast, Southern Sub‐Saharan Africa (1.03%) experienced an increase (Table 1, Figure 1C), highlighting regional inequities in stroke prevention and care.

**TABLE 1:** The age‐standardized death rates (ASMR) and cases (in hundreds) of HLIS in 1990 and 2021, and the temporal trends from 1990 to 2021.

Characteristics	1990		2021		EAPC(95%CI)
	Numbers (95%UI)	ASMR(95%UI)	Numbers (95%UI)	ASMR (95%UI)	
Global	6545.03 [2101.61–11038.93]	20.03 [6.28–34.56]	9361.92 [2995.35–16142.94]	11.38 [3.62–19.65]	−2.06 [−2.16 to −1.96]
Sex					
Female	3681.89 [1159.05–6342.09]	19.4 [6.04–33.8]	4748.09 [1488.93–8369.57]	10.12 [3.18–17.81]	−2.38 [−2.5 to −2.26]
Male	2863.15 [964.44–4747.49]	20.36 [6.41–34.84]	4613.82 [1506.42–7862.79]	12.81 [4.09–22.14]	−1.67 [−1.75 to −1.59]
Socio‐demographic index					
High SDI	1744.24 [549.28–2985.91]	15.79 [4.99–27.01]	1320.54 [378.18–2326.04]	5.2 [1.6–9.04]	−3.88 [−4.04 to −3.71]
High‐middle SDI	2651.52 [860.5–4406.59]	32.25 [10.21–54.87]	3189.49 [1019.87–5475.49]	16.45 [5.25–28.31]	−2.59 [−2.81 to −2.37]
Middle SDI	1302.86 [439.95–2211.7]	16.51 [5.16–28.69]	3031.64 [983.14–5280.49]	12.88 [4.08–22.66]	−0.84 [−0.92 to −0.76]
Low‐middle SDI	638.33 [211.87–1090.37]	13.45 [4.26–23.64]	1406.72 [452.99–2427.23]	11.61 [3.59–20.28]	−0.48 [−0.52 to −0.45]
Low SDI	196.42 [58.83–346.9]	11.68 [3.35–21.05]	402.37 [123.57–710.53]	10.44 [3.13–18.93]	−0.4 [−0.46 to −0.35]
Region					
Andean Latin America	14.17 [4.38–23.95]	7.9 [2.39–13.6]	25.38 [7.93–44.55]	4.54 [1.41–7.98]	−2.01 [−2.22 to −1.81]
Australasia	28.88 [9.03–49.61]	13.24 [4.05–22.87]	25.72 [7.35–46.07]	3.9 [1.13–6.95]	−4.14 [−4.26 to −4.01]
Caribbean	30.51 [9.51–53.07]	13.18 [4.05–23.18]	50.91 [15.35–88.38]	9.31 [2.81–16.18]	−1.07 [−1.15 to −0.99]
Central Asia	89.68 [29.67–151.44]	21.28 [6.86–36.6]	124.41 [39.94–212.49]	18.33 [5.66–32.17]	−0.94 [−1.22 to −0.67]
Central Europe	551.49 [179.87–928.09]	41.81 [13.42–71.24]	460.11 [141.18–787.38]	19.09 [5.94–32.5]	−2.87 [−3.02 to −2.72]
Central Latin America	65.8 [21.4–112.71]	9.7 [3.04–16.89]	115.93 [35.38–200.03]	4.95 [1.5–8.61]	−2.33 [−2.51 to −2.15]
Central Sub‐Saharan Africa	19.55 [5.55–35.13]	13.2 [3.53–24.38]	42.85 [12.6–81.23]	12.13 [3.23–23.58]	−0.43 [−0.49 to −0.37]
East Asia	1191.68 [390.27–2051.47]	18.22 [5.54–33.25]	3077.24 [950.3–5386.72]	15.69 [4.77–27.55]	−0.45 [−0.7 to −0.21]
Eastern Europe	1286.31 [425.29–2137.42]	51.87 [16.81–86.78]	1015.22 [340.52–1699.92]	28.15 [9.5–46.96]	−2.78 [−3.26 to −2.3]
Eastern Sub‐Saharan Africa	49.79 [14.9–88.26]	9.37 [2.72–17.44]	110.61 [34.16–200.46]	9.06 [2.65–16.7]	−0.2 [−0.28 to −0.12]
High‐income Asia Pacific	278.11 [88.24–484.98]	16.18 [4.95–28.4]	278 [78.88–506.21]	4.04 [1.19–7.22]	−4.82 [−4.99 to −4.65]
High‐income North America	330.22 [102.04–563.63]	8.91 [2.79–15.14]	296.76 [83.16–532.67]	4 [1.16–7.12]	−3.18 [−3.55 to −2.8]
North Africa and Middle East	368.7 [132.41–634.91]	27.77 [9.42–49.12]	732.58 [243.63–1270.81]	19.78 [6.25–35.16]	−1.09 [−1.13 to −1.05]
Oceania	2.23 [0.69–3.91]	11.8 [3.54–21.69]	4.97 [1.57–9.03]	9.86 [2.91–18.33]	−0.73 [−0.8 to −0.66]
South Asia	405.38 [131.97–722.35]	9.26 [2.82–16.67]	1009.78 [337.76–1781.9]	8.21 [2.6–14.56]	−0.55 [−0.66 to −0.44]
Southeast Asia	336.96 [109.35–581.63]	17.25 [5.28–30.52]	858.7 [278.21–1479.22]	16.13 [5.01–28.52]	−0.16 [−0.28 to −0.03]
Southern Latin America	63.46 [20.25–109.44]	15.36 [4.85–26.61]	52.64 [16.19–92.88]	5.7 [1.76–10.03]	−2.87 [−3.01 to −2.72]
Southern Sub‐Saharan Africa	22.35 [7.18–39.6]	10.18 [3.03–18.46]	58.49 [17.77–104.86]	13.31 [3.96–24.37]	1.03 [0.53 to 1.53]
Tropical Latin America	169.94 [57.12–281.76]	23.2 [7.46–39.21]	212.37 [70.4–363.52]	8.67 [2.85–14.92]	−3.03 [−3.16 to −2.9]
Western Europe	1129.65 [345.21–1946.87]	18.74 [5.78–32.22]	591.91 [171.13–1047.96]	4.71 [1.4–8.23]	−4.65 [−4.82 to −4.47]
Western Sub‐Saharan Africa	110.18 [31.96–197.91]	16 [4.53–29.13]	217.34 [68.84–373.74]	14.65 [4.43–25.39]	−0.27 [−0.37 to −0.18]

*Note*: HLIS: High LDL‐C‐related ischemic stroke; ASMR: Age‐standardized mortality rate, per 100,000 population; EAPC: Estimated annual percentage change; UI: Uncertainty interval; SDI: Socio‐demographic index; Numbers: Death cases (unit: hundreds).

**FIGURE 1:**
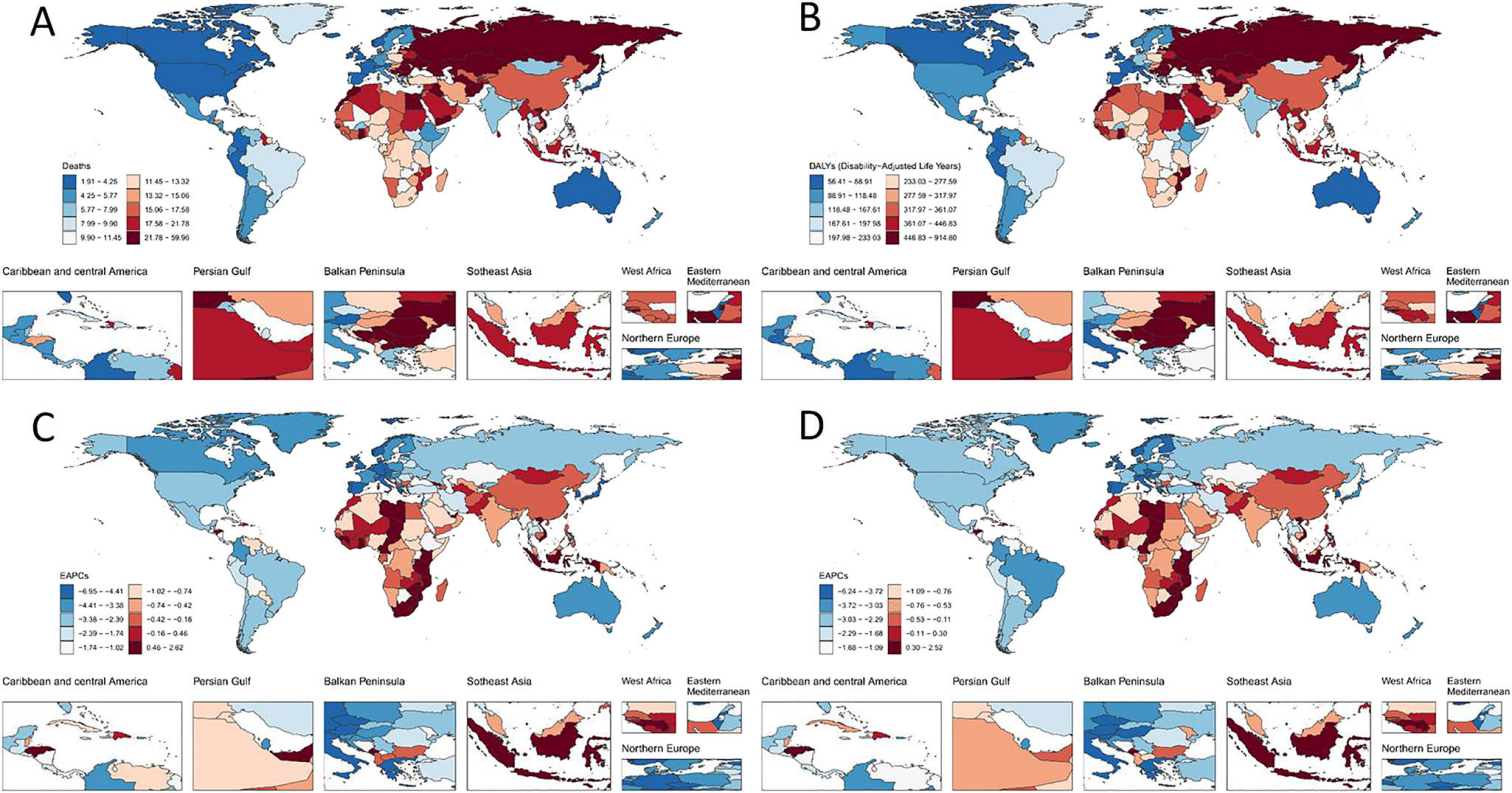
The age‐standardized rates and EAPCs of HLIS by countries in 2021 (A) Age‐standardized death rates; (B) Age‐standardized DALY rates in 2021. (C)EAPC for ASMR (D) EAPC for ASDR. HLIS, High LDL‐C‐related ischemic stroke; EAPC, Estimated annual percentage change; DALY, Disability‐adjusted life year; ASMR, Age‐standardized mortality rate; ASDR, Age‐standardized DALY rate; LDL‐C, Low‐density lipoprotein cholesterol.

Globally, HLIS‐related DALYs reached 20,977,424 (95% UI: 7,409,044–34,664,410) in 2021, marking a 44.55% rise since 1990 (Table 2). East Asia (7,056,799 DALYs) and Eastern Europe (1,990,872 DALYs) bore the highest burdens, potentially due to high dyslipidemia prevalence and uneven healthcare access, while Oceania (14,958 DALYs) and Australasia(45796) had the lowest (Table 2). The age‐standardized DALY rate (ASDR) was 246.42 per 100,000 (95% UI: 86.47–406.28), with Eastern Europe (565.86 per 100,000) and Central Asia (409.88 per 100,000) exhibiting the highest rates, and Australasia (80.37 per 100,000) and Andean Latin Americ (94.36 per 100,000) the lowest (Table 2, Figure 1B), underscoring the advantages of high‐SDI healthcare systems. Between 1990 and 2021, the ASDR declined at an EAPC of −1.72% (95% UI: −1.82 to −1.63), with Western Europe (−4.01%) and high‐income Asia Pacific (−3.84%) achieving the most significant improvements. Southern Sub‐Saharan Africa (0.61%) was the only region with an upward trend (Table 2, Figure 1D), emphasizing the need for enhanced stroke interventions in low‐resource settings.

**TABLE 2:** The age‐standardized DALYs rates (ASDR) and cases (in hundreds) of HLIS in 1990 and 2021, and the temporal trends from 1990 to 2021.

	1990		2021		EAPC(5%CI)
	Numbers (95%UI)	ASDR(95%UI)	Numbers (95%UI)	ASDR (95%UI)	
Global	145124.9 [51873.67–230681.98]	390.89 [133.81–632.33]	209774.24 [74090.44–346644.1]	246.42 [86.47–406.28]	−1.72 [−1.82 to −1.63]
Sex					
Female	75443.34 [26522.37–121742.9]	368.47 [126.36–599.97]	99374.89 [34355.36–164879.69]	215.42 [75.19–355.77]	−2.01 [−2.12 to −1.9]
Male	69681.55 [26087.46–109437.81]	412.18 [144.62–665]	110399.35 [39696.05–181764.9]	280.47 [98.55–469.59]	−1.43 [−1.51 to −1.35]
Socio‐demographic index					
High SDI	33154.48 [11402.56–53889.52]	299.12 [104.22–483.68]	26632.16 [9009.57–43796.8]	125.19 [44.83–199.95]	−3.08 [−3.23 to −2.92]
High‐middle SDI	56482.74 [20348.01–88930.81]	606.86 [209.65–974.04]	66091.5 [23207.67–108378.19]	337.2 [119.19–552.44]	−2.32 [−2.54 to −2.1]
Middle SDI	33537.92 [12524.84–53722.48]	345.38 [120.84–573.17]	71486.61 [25414.46–117761.16]	275.17 [95.54–458.71]	−0.79 [−0.84 to −0.73]
Low‐middle SDI	16406.67 [5908.78–26966.7]	284.26 [97.09–476.9]	34602.2 [12367.31–57299.42]	248.6 [83.94–421.23]	−0.46 [−0.49 to −0.43]
Low SDI	5308.17 [1709.95–8912.81]	251.39 [78.86–432.3]	10741.18 [3796.36–17992.95]	222.55 [72.33–383.74]	−0.48 [−0.54 to −0.43]
Region					
Andean Latin America	328.94 [116.48–525.54]	162.91 [54.58–266.37]	552.64 [192.96–919.14]	94.36 [32.48–157.4]	−1.97 [−2.18 to −1.76]
Australasia	523.24 [175.57–864.07]	228.3 [76.58–377.16]	457.96 [147.72–775.87]	80.37 [27.14–132.37]	−3.57 [−3.74 to −3.4]
Caribbean	642.8 [226.84–1070.79]	254.93 [87.27–429.84]	1031.33 [355–1736.72]	191.26 [66.14–322.08]	−0.86 [−0.94 to −0.78]
Central Asia	2311.39 [852.98–3649.43]	497.18 [177.75–802.78]	3252.73 [1202.06–5203.56]	409.88 [144.76–672.31]	−1.11 [−1.4 to −0.82]
Central Europe	10873.32 [3845.79–17375.11]	762.42 [267.29–1228.75]	8242.43 [2738.13–13592.72]	361.96 [124.88–587.3]	−2.74 [−2.88 to −2.6]
Central Latin America	1513.28 [564.44–2400.62]	189.89 [66.2–310.42]	2516.42 [903.26–4043.87]	102.57 [36.07–166.47]	−2.21 [−2.4 to −2.01]
Central Sub‐Saharan Africa	556.11 [178.43–946.03]	281.02 [84.64–497.77]	1177.8 [393.34–2071.91]	248.41 [76.38–453.71]	−0.55 [−0.6 to −0.49]
East Asia	31449.56 [11139.17–51518.66]	386.91 [130.76–648.43]	70567.99 [23852.57–117326.87]	333.23 [112.38–560.93]	−0.46 [−0.63 to −0.28]
Eastern Europe	26510.25 [9726.28–41620.05]	984.13 [353.54–1560.85]	19908.72 [7121.96–31768.69]	565.86 [206.45–894.67]	−2.57 [−3.04 to −2.1]
Eastern Sub‐Saharan Africa	1406.01 [453.96–2417.3]	205.12 [63.36–366.3]	3071.57 [1042.52–5276.09]	196.28 [62.89–346.62]	−0.25 [−0.31 to −0.18]
High‐income Asia Pacific	5786.12 [1992.37–9523.9]	305.43 [104.08–507.65]	5157.49 [1638.59–8819.15]	103.65 [36.02–167.03]	−3.84 [−4 to −3.68]
High‐income North America	6756.24 [2379.81–10933.79]	188.59 [67.86–302.78]	6600.66 [2213.81–11099.53]	102.78 [35.97–168.01]	−2.43 [−2.71 to −2.15]
North Africa and Middle East	9322.93 [3613.93–15187.39]	574.64 [212.27–970.58]	18316.71 [6940.29–29396.69]	411.22 [145.05–685.05]	−1.11 [−1.14 to −1.08]
Oceania	69.3 [25.64–112.88]	259.85 [87.29–447.32]	149.58 [56.39–251.75]	218.38 [72.49–383.36]	−0.7 [−0.76 to −0.64]
South Asia	10716.91 [3858.91–18159.34]	198.11 [67.03–341.13]	24425.38 [8963.39–41158.22]	172.3 [61.02–293.31]	−0.62 [−0.72 to −0.53]
Southeast Asia	8928.42 [3249.65–14357.59]	370.76 [125.67–619.06]	21659.77 [7775.57–35482.49]	347.11 [119.88–576.98]	−0.18 [−0.26 to −0.09]
Southern Latin America	1299.84 [452.78–2112.03]	292.66 [100.96–479.41]	1078.14 [367.2–1767.65]	121.56 [42.26–197.7]	−2.65 [−2.77 to −2.52]
Southern Sub‐Saharan Africa	610.52 [226.05–994.47]	232.27 [80.01–395.24]	1420.32 [488.6–2368.03]	269.14 [85.73–470.81]	0.61 [0.18 to 1.04]
Tropical Latin America	3827.02 [1438.19–5966.06]	446.94 [158.06v721.46]	4347.85 [1612.21–6933.07]	171.85 [62.72–276.09]	−3.09 [−3.26 to −2.92]
Western Europe	18864.12 [6244.08–31165.22]	316.62 [107.58–517.56]	9998.35 [3207.37–16952.77]	96.36 [33.06–158.94]	−4.01 [−4.21 to −3.82]
Western Sub‐Saharan Africa	2828.57 [927.38–4759.15]	339.86 [105.72–583.82]	5840.38 [2083.27–9740.26]	310.14 [102.31–526.16]	−0.29 [−0.39 to −0.19]

*Note*: HLIS: High LDL‐C‐related ischemic stroke; DALY: Disability‐adjusted life year; ASDR: Age‐standardized; DALY rate, per 100,000 population; EAPC: Estimated annual percentage change; UI: Uncertainty interval; SDI: Socio‐demographic index; Numbers: DALY cases (unit: hundreds).

### 3.2 Joinpoint Regression Analysis

Based on GBD 1990–2021 data, global mortality of HLIS exhibited significant declining trends, though with notable disparities across SDI levels. The global AAPC in mortality was −1.80%, with the steepest decline in high‐SDI regions (AAPC = −3.52%), and limited improvement in low‐SDI regions (AAPC = −0.33%) (Figure S1). The sharpest mortality reduction occurred during 2003–2006 (APC = −3.50%), while the sustained acceleration in high‐SDI regions (2013–2021, APC = −1.89%) reflects advancements in chronic disease management.

Similarly, global HLDLSD‐related DALYs declined at an AAPC of −1.47%, with the most substantial progress in high‐SDI regions [(AAPC = −2.77%) and slower gains in low‐SDI regions (AAPC = −0.37%) (Figure S2). The steepest global DALY reduction occurred during 2004–2008 (APC = −3.48%), likely linked to Millennium Development Goals (MDGs)‐driven infectious disease control, while high‐middle SDI regions achieved their fastest decline earlier (2003–2012, APC = −3.80%). Notably, after 2014, high‐SDI regions experienced a slowdown in DALY reduction (APC = −1.12%) (Figure S2).

### 3.3 Association Between SDI and HLIS Burden

Our analysis revealed a weak negative correlation (r = −0.131, *p* < 0.001) between SDI and ASMR of HLIS across 21 global regions from 1990 to 2021 (Figure 2A). Regions characterized by elevated SDI values, such as high‐income North America and Western Europe, typically demonstrated reduced mortality rates; conversely, areas with low SDI, notably Sub‐Saharan Africa, encountered a substantially greater disease burden. A stronger negative correlation was observed between SDI and ASDR (r = −0.175, *p* < 0.001), with Eastern Europe having the highest DALY rate in 2021 (565.86 per 100,000) and high‐income Asia Pacific the lowest (103.65 per 100,000). Although the global ASDR declined overall (390.89 → 246.42 per 100,000), regions such as Central Asia and Eastern Europe remained disproportionately affected (Figure 2B).

**FIGURE 2:**
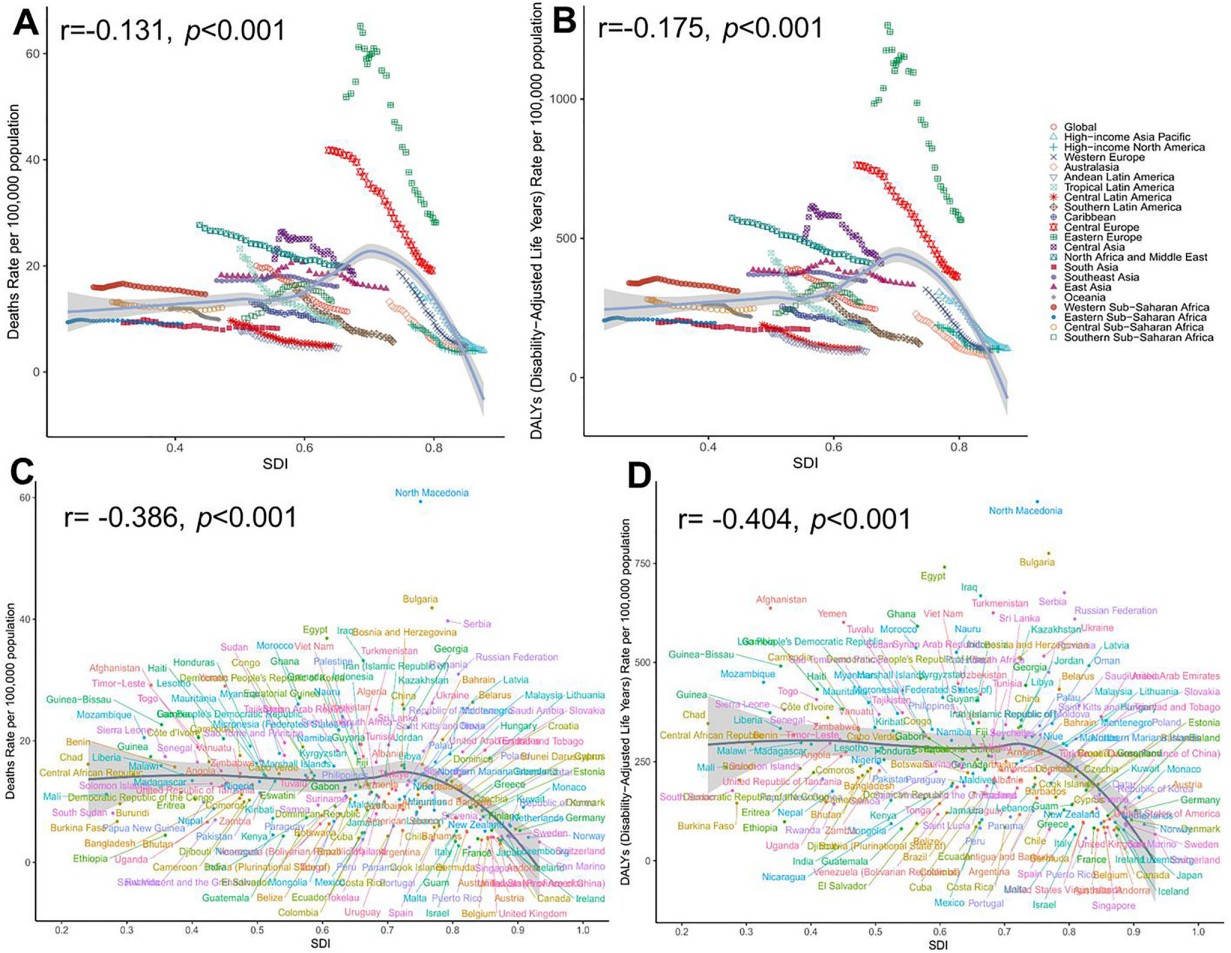
Correlation analysis of ASMR and ASDR due to HLIS with the Socio‐Demographic Index across global regions and 204 countries. A: Correlation between ASMR and SDI across 21 regions. B: Correlation between ASDR and SDI across 21 regions. C: Correlation between ASMR and SDI across 204 countries. D: Correlation between ASDR and SDI across 204 countries. ASMR: Age‐standardized mortality rate; ASDR: Age‐standardized DALY rate. DALY: Disability‐Adjusted Life Year. HLIS, High LDL‐C‐related ischemic stroke; ASMR, Age‐standardized mortality rate; ASDR, Age‐standardized DALY rate; DALY, Disability‐adjusted life year; SDI, Socio‐demographic index; LDL‐C, Low‐density lipoprotein cholesterol.

At the national level (GBD 2021, 204 countries/territories), the ASMR of HLDLSD showed a significant inverse correlation with SDI (r = −0.386, *p* < 0.001) (Figure 2C). The highest mortality rates were observed in Eastern European nations (North Macedonia: 59.37 per 100,000; Bulgaria: 41.87 per 100,000) and North African countries (Egypt: 36.87 per 100,000), while high‐income countries (Singapore: 1.92 per 100,000; Japan: 3.68 per 100,000) reported the lowest rates (Table S4). Notably, Puerto Rico (2.50 per 100,000) performed better than expected, whereas Nauru (21.78 per 100,000) emerged as an outlier among high‐SDI countries, suggesting that its obesity epidemic may counteract socioeconomic advantages.

Similarly, SDI and DALY rates exhibited a strong negative correlation (r = −0.404, p < 0.001) (Figure 2D). High‐SDI countries (Singapore: 70.0 per 100,000; Australia: 77.9 per 100,000) had substantially lower DALY rates than low‐SDI nations (North Macedonia: 905.7 per 100,000; Bulgaria: 775.2 per 100,000) (Table S5). Of particular concern, some middle‐SDI countries (e.g., Egypt: 740.8 per 100,000) displayed disproportionately high disease burdens, indicating that factors beyond SDI—such as healthcare resource allocation and dietary patterns—play critical roles.

### 3.4 Age‐ and Sex‐Specific Disparities

Our results demonstrate substantial variations in the mortality burden attributable to HLIS, stratified by both age and sex. Women aged 85+ years exhibited substantially higher mortality than their male counterparts (e.g., 95+ age group: 25,468 deaths in women vs. 7,293 in men), with the mortality rate in women aged 95+ reaching 646.67 per 100,000—1.34 times higher than in men. Conversely, middle‐aged men (55–64 years) had significantly higher mortality than women (e.g., 60–64 years: 29.03 per 100,000 in men vs. 16.13 in women, sex ratio 1.8). Although younger adults (25–39 years) had low absolute death counts, they displayed the highest attributable fraction to high LDL‐C (56.3% in men aged 35–39), underscoring the critical risk of early‐onset atherosclerosis. From 1990–2021, the global ASMR remained higher in men (2021: 12.81 per 100,000) than in women (10.12 per 100,000), though both sexes showed declining trends (Figure 3A, Table S1).

**FIGURE 3:**
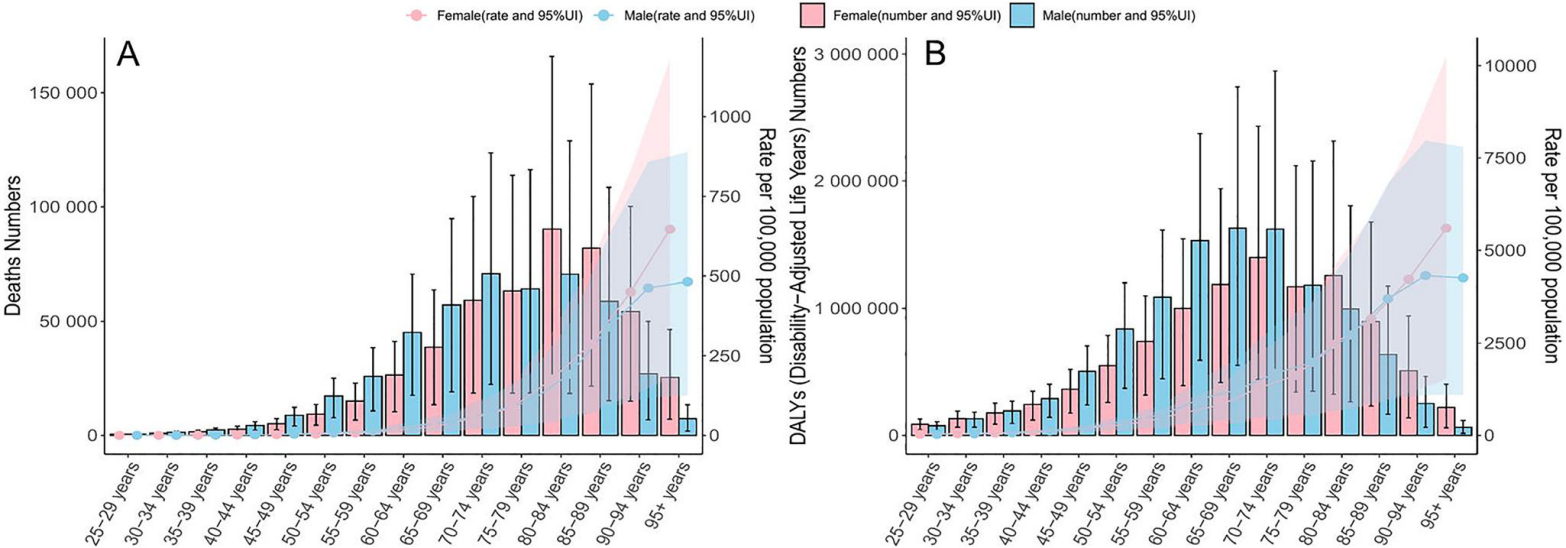
Age and sex specific rate and numbers of global HLIS death (A) and DALYs (B) in 2021. HLIS, High LDL‐C‐related ischemic stroke; DALY, Disability‐adjusted life year; LDL‐C, Low‐density lipoprotein cholesterol.

The age‐specific burden of DALYs exhibited an inverted U‐shaped trajectory, reaching its apex among males in the 65–69 year cohort (1,628,942.60 DALYs) and among females in the 70–74 year group (1,398,468.52 DALYs). However, age‐standardized DALY rates increased continuously with age, with women aged 95+ (5599.26 per 100,000) experiencing a 31.6% higher rate than men (4255.08 per 100,000)—likely due to female longevity and postmenopausal metabolic changes. Young and middle‐aged men (25–49 years) had higher absolute DALYs (e.g., 35–39 years: 192,961.61 in men vs. 177,914.62 in women), and the attributable fraction to high LDL‐C peaked in this group (55.3% in men, 56.2% in women), declining to 22%–24% in the elderly. Over 1990–2021, men consistently had higher DALY rates than women (2021: 280.47 vs. 215.42 per 100,000), though both sexes showed significant improvements (Figure 3B, Table S1).

### 3.5 Decomposition Analysis

Between 1990 and 2021, the worldwide mortality burden attributable to HLIS rose by 281,688 cases, primarily driven by population aging (contributing 96.3%, +271,191 cases) and population growth (172.7%, +486,506 cases), which were partially offset by epidemiological improvements (−169.0%, −476,010 cases) (Figure 4A, Table S2). High‐SDI regions experienced a reduction of 42,370 deaths, largely attributable to epidemiological improvements (+438.9%, −185,946 cases) that completely counterbalanced the negative effects of aging (+82,430 cases) and population growth (+61,146 cases). Conversely, regions characterized by low SDI values witnessed a rise of 20,595 fatalities, mainly driven by population growth (+123.2%, +25,379 cases) (Figure 4A, Table S2). Sex‐specific analysis revealed that male deaths increased by 175,067 cases, with aging (+75.8%) and population growth (+126.7%) being the primary contributors, while female deaths rose by 106,620 cases, where aging (+55.8%) and population growth (+130.2%) played more prominent roles, though epidemiological improvements (−86.0%) showed stronger mitigating effects (Figure 4B,C; Table S2).

**FIGURE 4:**
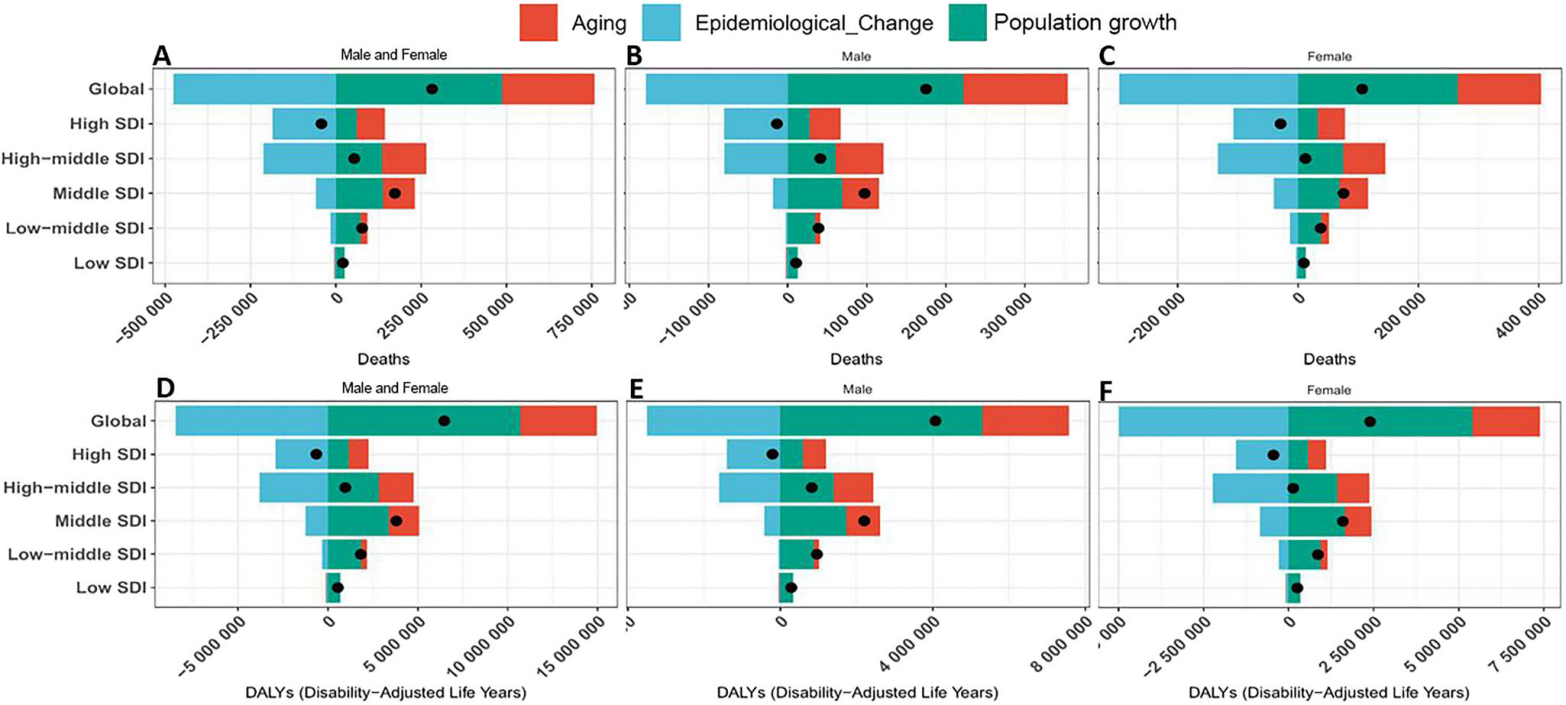
Decomposition of drivers for HLIS burden changes across global and SDI regions, 1990–2021. A‐C: Deaths; D‐F: Disability‐adjusted life years (DALYs);A/D: Both sexes; B/E: Male;C/F: Female. Light blue = aging effect, green = epidemiological change effect, red = population growth effect, “Both” = combined effects; Black dots indicate total change (sum of all components); Bar length to the right denotes positive contribution, left denotes negative contribution. HLIS, High LDL‐C‐related ischemic stroke; SDI, Socio‐demographic index; DALY, Disability‐adjusted life year; LDL‐C, Low‐density lipoprotein cholesterol.

Globally, DALYs increased by 6.46 million, with population growth (+165.6%, +10.71 million) and aging (+65.5%, +4.24 million) serving as the main drivers, while epidemiological improvements (−131.2%) partially offset this potential growth (Figure 4D, Table S2). High‐SDI regions achieved a reduction of 650,000 DALYs, where epidemiological improvements (+446.4%) fully neutralized the impacts of aging (−169.2%) and population growth (−177.2%). Conversely, middle‐SDI regions experienced an increase of 960,000 DALYs, as population growth (+294.2%) and aging (+202.6%) outweighed the benefits of epidemiological improvements (−396.7%) (Figure 4D, Table S2). Significant sex differences were observed: male DALYs increased by 4.07 million, predominantly due to population growth (+130.2%), while female DALYs rose by 2.39 million, mainly driven by population growth (+225.8%) and aging (+82.9%), though epidemiological improvements (−208.6%) demonstrated more pronounced mitigating effects (Figures 4D, 4E; Table S2). In low‐SDI regions, DALY increases were primarily fueled by population growth (males: +91.9%, females: +109.5%), reflecting the inadequacy of healthcare systems in coping with demographic pressures.

### 3.6 Country‐Level Analysis

Global ASMR of HLIS exhibited substantial regional disparities. Eastern European countries demonstrated the highest ASMR in 1990, including Serbia (72.20, 95% UI 23.43–125.00), but showed remarkable reductions by 2021, as seen in Czechia (EAPC −5.94, 95% CI −6.37 to −5.52) and Hungary (EAPC −4.10, 95% CI −4.31 to −3.90). East Asia displayed pronounced heterogeneity in 2021 ASMR, with Japan (3.68, 95% UI 1.09–6.51) and Singapore (1.92, 95% UI 0.56–3.39) having the world's lowest rates, while China (15.93, 95% UI 4.83–28.08) and North Korea (18.98, 95% UI 6.17–34.00) remained elevated (Table S4, Figure 2A,C). Among Southeast Asian nations, Vietnam (22.95, 95% UI 6.89–41.81; EAPC 0.52, 95% CI 0.39–0.66) and Indonesia (19.43, 95% UI 5.62–35.48; EAPC 1.10, 95% CI 0.95–1.24) showed increasing trends, while Thailand (8.00, 95% UI 2.63–14.15; EAPC −2.30, 95% CI −2.57 to −2.03) achieved significant reductions. Sub‐Saharan African countries like Ghana (27.06, 95% UI 7.82–47.80) and Gambia (20.54, 95% UI 5.88–37.97) maintained persistently high burdens, with several nations showing significant EAPC increases (e.g., Zimbabwe 1.71, 95% CI 1.16–2.28) (Table S4, Figure 2A,C), indicating healthcare resource deficiencies and delayed disease control measures.

DALY rate trends revealed that while Eastern European nations remained high (e.g., Serbia 675.98, 95% UI 214.65–1134.42), they achieved substantial declines (EAPC −2.40, 95% CI −2.66 to −2.15). High‐income countries such as Singapore (70.03, 95% UI 25.02–110.88; EAPC −5.70, 95% CI −5.87 to −5.54) and Japan (97.00, 95% UI 34.01–155.52; EAPC −3.53, 95% CI −3.69 to −3.38) maintained the lowest burdens. China's DALY rate decreased from 385.65 (95% UI 129.83–647.51) to 335.59 (95% UI 112.75–566.25; EAPC −0.42, 95% CI −0.60 to −0.24), whereas Indonesia showed the most pronounced Southeast Asian increase from 328.53 (95% UI 111.41–576.55) to 404.90 (95% UI 132.32–697.84; EAPC 0.74, 95% CI 0.62–0.86) (Table S5, Figures 2B and 2D). Sub‐Saharan Africa (e.g., Ghana 591.44, 95% UI 189.66–1004.62) and South Asia (e.g., Afghanistan 637.15, 95% UI 226.4–1127.34) sustained elevated burdens, with multiple countries displaying positive EAPCs (Lesotho 2.50, 95% CI 2.04–2.96; Mozambique 1.31, 95% CI 1.12–1.49) (Table S5, Figures 2B and 2D), reflecting overlapping infectious/chronic diseases and fragile health systems. The global DALY rate heterogeneity underscores that economic development alone isn't deterministic, necessitating comprehensive interventions for high‐burden nations.

### 3.7 Health Inequality Analysis

The GBD‐based health inequality analysis revealed a significant reversal in the socioeconomic gradient of global HLIS burden from 1990 to 2021. In 1990, the slope index of inequality (SII) showed a positive mortality gradient (5.52, 95% CI: 2.03–9.00), which transitioned to a negative gradient by 2021 (−6.12, 95% CI: −8.55 to −3.69), indicating a shift in disease burden from high‐income to low‐income populations (Figure 5A). This trend was more pronounced for DALYs (SII declined from 61.14 to −136.15) (Figure 5B). The concentration index (CII) corroborated this reversal: mortality CII decreased from 0.08 (0.05–0.12) to −0.09 (−0.13 to −0.04) (Figure 5C), while DALYs' CII declined from 0.05 (0.01–0.08) to −0.10 (−0.14 to −0.06) (Figure 5D). This shift reflects widening disparities between high‐income nations' medical advancements (e.g., thrombolysis uptake) and persistent healthcare resource shortages in low‐income countries.

**FIGURE 5:**
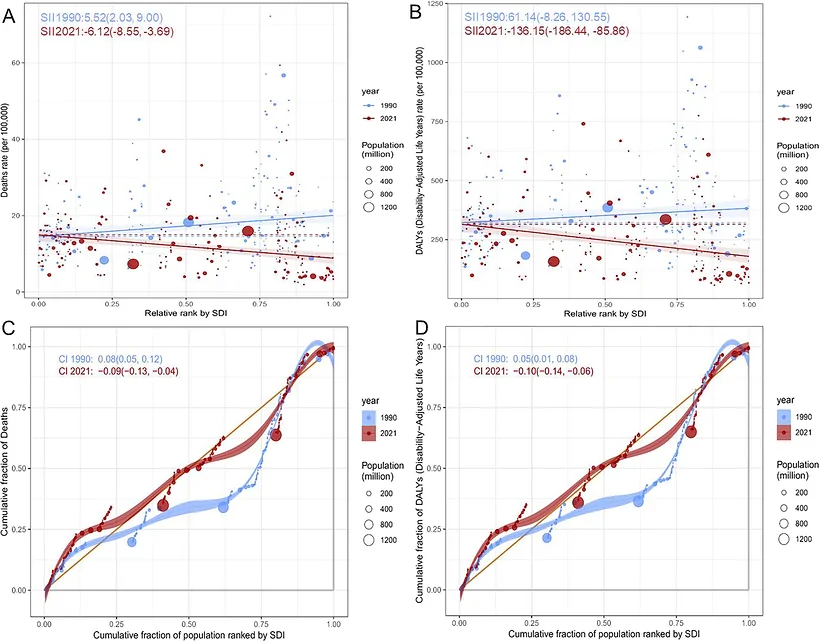
Rankings of the Slope Index of Inequality (SII) and Concentration Index (CI) for the age‐standardized mortality rate (ASMR) and disability‐adjusted life years (DALYs) of high LDL‐C‐related ischemic stroke (HLIS) in 1990 and 2021 globally by Socio‐demographic Index (SDI) quintiles. (A) SII for HLIS burden (males); (B) SII for HLIS burden (females); (C) CI for HLIS burden (males); (D) CI for HLIS burden (females). CI, Concentration Index; SII, Slope Index of Inequality; ASMR, Age‐standardized mortality rate; DALY, Disability‐adjusted life year; HLIS, High LDL‐C‐related ischemic stroke; SDI, Socio‐demographic index; LDL‐C, Low‐density lipoprotein cholesterol.

### 3.8 Age‐Period‐Cohort Analysis

Both mortality and DALY rates for HLIS exhibited exponential increases with age (Figures 6A,6D). The 27.5‐year age group showed the lowest mortality (0.42 per 100,000, 95% CI: 0.37–0.48) (Table S3) and DALY rate (49.07 per 100,000, 95%CI: 46.94–51.29) (Table S3), while the 97.5‐year group demonstrated a dramatic rise to 362.85 per 100,000 (95% CI: 351.08–375.00) (Table S3, Figure S3B) for mortality and 3269.62 (95% CI: 3149.54–3394.28) (Table S3, Figure S4B) for DALYs.

**FIGURE 6:**
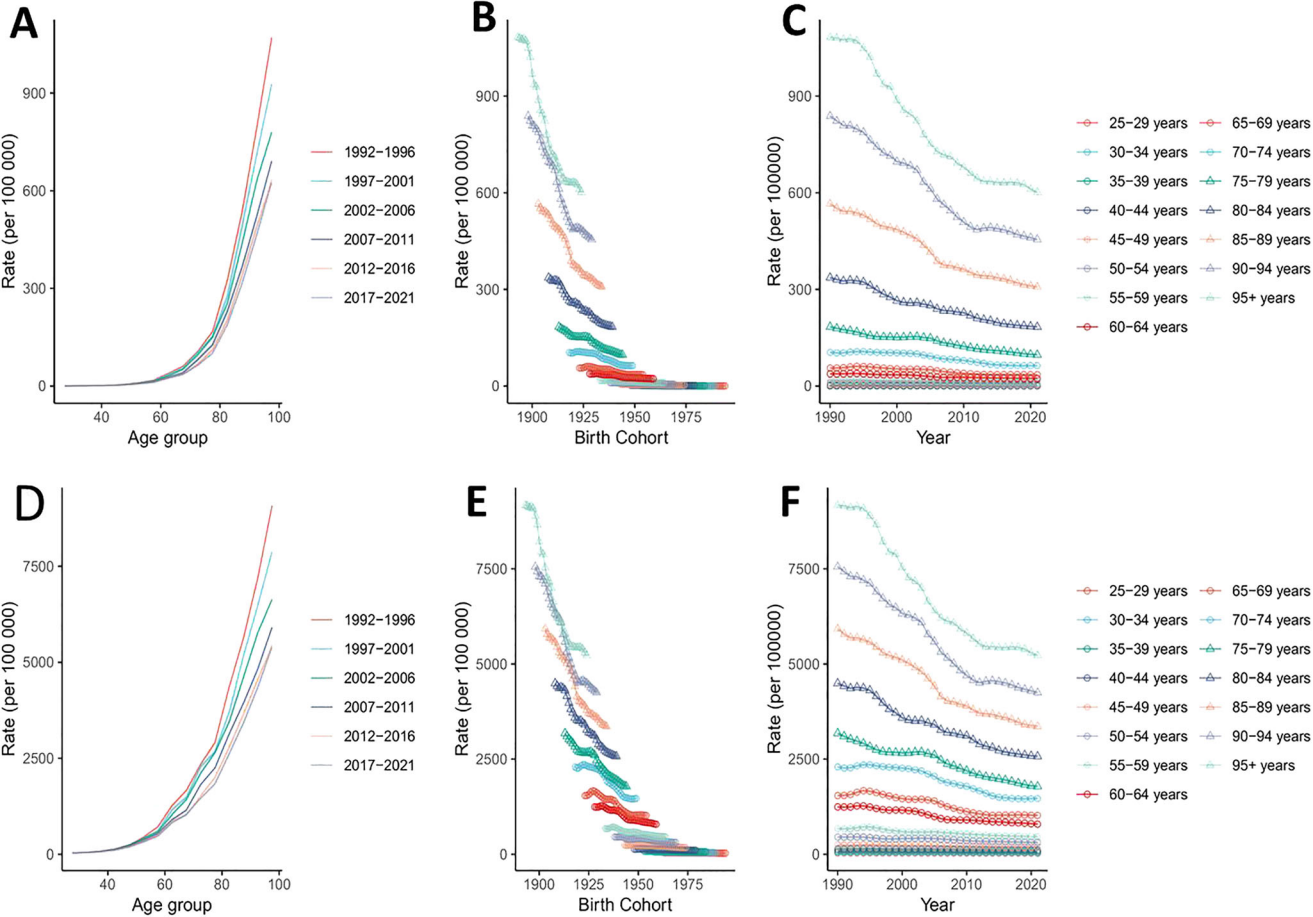
Results of age‐period‐cohort model analysis for HLIS mortality in global, 1990–2021. (A) Age‐specific mortality rates by period. (B) Cohort‐specific ASMR by age group. (C) Age‐specific mortality rates by year. (D) Age‐specific DALY rates by period. (E) Cohort‐specific ASDR by age group; (F) Age‐specific DALY rates by year. HLIS, High LDL‐C‐related ischemic stroke; ASMR, Age‐standardized mortality rate; DALY, Disability‐adjusted life year; ASDR, Age‐standardized DALY rate; LDL‐C, Low‐density lipoprotein cholesterol.

Between 1992 and 2021, a consistent downward trajectory in both mortality and disability‐adjusted life year (DALY) rates was observed across all age demographics (Table S3, Figures S3C, S3F). Using 2004.5 as the reference period (RR = 1), mortality risk increased by 16.5% during 1994.5–2004.5 but decreased significantly by 27.2% from 2004 to 2019 (2019.5 RR = 0.728). However, the rate of decline slowed in recent years (2014.5–2019.5), with an annual reduction of approximately 1.2% (Table S3, Figure S3C). A similar downward trend was observed for DALY rates, with the age‐standardized RR decreasing from 1.13 in 1994.5 to 0.76 in 2019.5 (Table S4, Figure S4C). Local drift analysis revealed negative drifts across all age groups (annual change: −1.25% to −2.51%), with the most pronounced declines in middle‐aged groups (42.5–62.5 years: −1.57% to −2.16%). Older age groups (≥72.5 years) exhibited a U‐shaped drift pattern (lowest at 82.5 years: −2.09%), and the net drift was −1.95% per year (95% CI: −2.04 to −1.87) (Table S3, Figures S4A, S5A).

Significant birth cohort effects were observed: using the 1942 cohort as reference (RR = 1), earlier cohorts (1897–1937) showed progressive risk reduction (1897 cohort RR = 2.98 → 1937 cohort RR = 1.18), while later‐born cohorts (post‐1942) continued to demonstrate declining risk (1947 cohort RR = 0.88 → 1992 cohort RR = 0.46) (Table S3, Figure 6B). However, the rate of decline slowed in recent decades (1987–1992 cohorts: only 11% reduction in RR) (Figure S4D). DALY rates similarly reflected lower burdens in later‐born cohorts (Table S3, Figure 6E); for example, the DALY rate for 30–34‐year‐olds decreased from 52.70 in the 1958 cohort to 43.29 in the 1989 cohort (Table S3, Figure S4D).

## 4 Discussion

The present investigation conducted a comprehensive evaluation of the epidemiological profiles associated with HLIS from 1990 to 2021 using data from the GBD study, unveiling a strong association between disease burden and socioeconomic development levels. The findings indicate that although the global ASMR and DALY rate exhibited a declining trend, significant regional disparities persisted: high SDI regions—characterized by well‐established healthcare systems—achieved the most substantial improvements, whereas low‐SDI regions (particularly those in sub‐Saharan Africa) experienced a continuous increase in disease burden, a pattern that may reflect global inequities in the allocation of health resources. The escalating absolute case counts are primarily attributable to demographic shifts, specifically population aging and expansion, a trend that is markedly accentuated in areas characterized by insufficient healthcare infrastructure. This study also revealed significant sex‐ and age‐related disparities: older women had higher mortality rates, middle‐aged men bore a heavier disease burden, and younger populations exhibited the highest LDL‐C‐attributable risk. Notably, birth cohort analysis demonstrated a continuous decline in lifetime risk among later‐born generations; Nevertheless, the pace of advancement has decelerated in recent years, suggesting that the current epidemic of non‐communicable diseases may be offsetting some gains in chronic disease prevention and control. These results underscore the imperative for region‐specific lipid management protocols adapted to varying developmental stages, while urging the global community to address the escalating disparity in stroke burden as a paramount public health priority.

Our study further delineated the global epidemiological characteristics of HLIS and its intimate association with socioeconomic development levels. The analysis showed that although the global ASMR and DALY rate of HLIS exhibited an overall declining trend from 1990 to 2021, marked regional disparities persisted. High‐SDI regions, particularly those in high‐income Asia‐Pacific and Western Europe, achieved the most rapid improvements, possibly facilitated by well‐established chronic disease management systems (Li et al. 2025; Meng et al. 2025). In contrast, Southern Sub‐Saharan Africa was the only region where the disease burden showed an increasing trend. Notably, while Eastern Europe still has the highest global disease burden, its notable decline is consistent with prior reports that the implementation of structured cardiovascular risk management programs in primary healthcare can substantially reduce disease burden even in economically constrained settings (Collins et al. 2020; Movsisyan et al. 2020). Because the present analysis did not directly evaluate intervention effects or resource allocation, this interpretation is proposed as a plausible mechanism informed by previous literature rather than a conclusion established by our data; likewise, the evolving distribution of disease burden is compatible with—but does not by itself demonstrate—a critical influence of inequitable health resource allocation on stroke prevention and control outcomes. Further analysis revealed more complex heterogeneity in disease burden at the national level. Considerable intra‐regional variations were observed in East Asia: Japan and Singapore have achieved the lowest global disease burden, whereas some Southeast Asian and sub‐Saharan African countries continue to experience rising trends. A pressing issue is that specific nations with middle SDI values shoulder a disease burden markedly excessive compared to their stage of economic progression, which underscores the significant influence of factors beyond GDP, such as the robustness of healthcare systems and the effectiveness of risk factor control. Several literature‐supported factors may contribute to this discordance, although none were directly measured in the present study. First, global pooled analyses indicate that the epicenter of non‐optimal cholesterol has shifted from high‐income Western countries toward low‐ and middle‐income regions, including North Africa, the Middle East, and Southeast Asia, implying that improvements in population lipid profiles have lagged behind economic development in these settings (Taddei et al. 2020). Second, the coverage of lipid‐lowering therapy remains low in many such countries: nationally representative surveys show that only a small fraction of eligible individuals in low‐ and middle‐income countries receive statins for primary or secondary prevention (Marcus et al. 2022), and the availability and affordability of cardiovascular medicines are markedly lower than in high‐income countries (Khatib et al. 2016). Third, constrained healthcare accessibility and limited primary care capacity may impede the timely detection and sustained management of hypercholesterolemia, while region‐specific risk profiles—including smoking, obesity, and dietary patterns high in saturated fat and salt—may further amplify the LDL‐C‐related stroke burden (Wu et al. 2024, NCD Risk Factor Collaboration (NCD‐RisC) 2024). These considerations are offered as potential explanations derived from external evidence and warrant confirmation in future country‐specific investigations. Joinpoint regression analysis indicated that high‐SDI regions experienced the most rapid improvements in the early 2000s, but the pace of progress has slowed in recent years, suggesting that the growing burden of non‐communicable diseases may limit further gains. These findings emphasize the necessity of moving beyond a purely economic development perspective and adopting tailored, comprehensive interventions to address region‐specific challenges.

Our analysis further revealed a pronounced inverse relationship between the SDI and the burden attributable to HLIS. While the global assessment indicated only a modest negative correlation between SDI and ASMR, this linkage became markedly more robust when examined at the national scale; specifically, nations characterized by higher SDI values consistently demonstrated substantially reduced disease burdens. Notably, certain middle‐SDI countries (e.g., Egypt) had substantially higher disease burdens than predicted by their economic development levels. Because our analysis is descriptive, it cannot directly identify the drivers of these outliers; nevertheless, previous literature offers plausible explanations. Dyslipidemia prevalence remains high in many middle‐SDI settings while LDL‐C control and statin coverage are inadequate (Taddei et al. 2020; Marcus et al. 2022), and primary healthcare systems may lack the capacity to deliver sustained lipid management (Khatib et al. 2016). Conversely, high‐SDI outliers such as Nauru indicate that favorable socioeconomic conditions do not invariably translate into a low disease burden; previous surveys have documented an exceptionally high prevalence of obesity and diabetes in Pacific Island nations (NCD Risk Factor Collaboration (NCD‐RisC) 2024), which may plausibly counteract their socioeconomic advantages. We emphasize that these mechanisms—including lifestyle factors and health resource allocation (Wu et al. 2024)—are hypotheses derived from external literature rather than findings of the present study, and country‐level heterogeneity in diet, smoking, and healthcare delivery warrants dedicated national investigations. Despite their relatively high SDI levels, Eastern European countries maintain the world's highest DALY burden, highlighting the presence of region‐specific risk factors (Movsisyan et al. 2020). These findings indicate that strategies for stroke and LDL‐C control must account for the complex interplay between socioeconomic factors and local risk profiles. Distinct age‐ and sex‐specific patterns were also identified: elderly women (≥85 years) had higher mortality rates than their male counterparts, which may be attributable to women's longer life expectancy and postmenopausal metabolic alterations (Stachowiak et al. 2015). In contrast, middle‐aged men (55–64 years) bore a disproportionately high disease burden, reflecting sex‐specific risk exposures (Betai et al. 2024). Subsequent decomposition analyses identified population aging and expansion as the predominant factors propelling the worldwide rise in ischemic stroke mortality and DALYs, an effect that is particularly pronounced in low‐SDI regions where fragile health systems struggle to accommodate demographic transitions (Stafford et al. 2023). It should be noted that the decomposition analysis partitions changes in burden into three statistical components—population aging, population growth, and epidemiological change (residual changes in age‐specific rates)—and does not directly incorporate indicators of healthcare system capacity or risk factor management. Within this framework, the favorable epidemiological‐change component in high‐SDI regions was large enough to fully offset the demographic contributions, whereas in low‐SDI regions demographic pressures dominated, resulting in persistently rising absolute disease burdens. Whether this favorable component reflects better risk factor control or healthcare delivery cannot be determined from the decomposition itself; nevertheless, prior studies documenting wider statin coverage and more established chronic disease management in high‐SDI settings support this as a plausible explanation (Li et al. 2025; Meng et al. 2025; Marcus et al. 2022). These results underscore the need for age‐ and sex‐tailored interventions, as well as the critical importance of strengthening primary prevention and health infrastructure in resource‐limited settings.

Our analysis of health inequalities uncovered a striking reversal in the socioeconomic gradient of the global HLIS burden between 1990 and 2021. The findings demonstrate a distinct shift in disease burden from high‐income to low‐income populations, a transition that is particularly pronounced when measured by DALYs. The present study describes socioeconomic gradients and their temporal changes but did not directly measure access to acute stroke care or the distribution of health resources. Nevertheless, the widening disparity is compatible with external evidence documenting pronounced global inequities in the availability of reperfusion services: access to intravenous thrombolysis and mechanical thrombectomy is concentrated in high‐income countries and remains scarce in low‐income settings (Kim et al. 2024). Unequal access to such medical technologies, together with shortages of health resources, may therefore be potential contributors to—rather than established causes of—the observed divergence. Importantly, dual validation using both the slope index of inequality (SII) and concentration index (CII) not only confirmed the robustness of this epidemiological transition but also underscored the systemic inequities in global stroke prevention frameworks, highlighting the urgent need for more equitable international allocation of medical resources. Age‐period‐cohort analysis elucidated several key epidemiological characteristics of the HLIS burden: First, disease burden increases exponentially with advancing age. Although mortality and DALY rates have declined across all age groups, the rate of improvement has slowed in recent years. Second, birth cohort analysis revealed a progressive reduction in lifetime risk among later‐born generations; however, the magnitude of this risk reduction was markedly smaller for the 1987–1992 birth cohort. Third, local drift analysis showed the most substantial improvements in middle‐aged groups (42.5–62.5 years), whereas elderly populations exhibited a U‐shaped temporal pattern. Because APC modeling partitions temporal variation into age, period, and cohort components without directly measuring their underlying drivers, these patterns cannot, by themselves, establish causal mechanisms. They are, however, consistent with hypotheses advanced in the literature: the declining period and cohort risks align temporally with decades of improvement in medical care and risk factor control (Feigin et al. 2024; Kisa and Kisa 2021); the attenuated risk reduction in the 1987–1992 birth cohort may plausibly reflect the rising prevalence of obesity, dyslipidemia, and other metabolic risk factors among younger generations documented in global analyses (Kaneko et al. 2021; Taddei et al. 2020; NCD Risk Factor Collaboration (NCD‐RisC) 2024), and the U‐shaped local drift among older adults may reflect longer cumulative exposure to historical risk environments and increasing multimorbidity with advancing age (Stachowiak et al. 2015). These interpretations are proposed as hypotheses to be tested in future studies incorporating direct measures of treatment uptake and risk factor trajectories. The findings provide crucial evidence for the development of targeted intervention strategies tailored to specific demographic and temporal risk patterns.

This study has several important limitations that warrant consideration when interpreting the global burden patterns of HLIS. First, HLIS is a modeled outcome derived from the GBD 2021 comparative risk assessment framework, rather than a prospectively collected, clinically adjudicated diagnosis of ischemic stroke directly attributable to high LDL‑C. Second, the attribution of ischemic stroke burden to elevated LDL‑C depends on predefined assumptions regarding population exposure distributions and pooled relative risk estimates from meta‑analyses, which may introduce systematic uncertainty. Third, the independent effects of LDL‑C cannot be fully disentangled from those of coexisting cardiometabolic conditions, including hypertension, diabetes mellitus, and atrial fibrillation, all of which share overlapping pathophysiological pathways with ischemic stroke. Fourth, residual confounding and complex risk factor interactions may distort burden estimates, especially in populations with a high prevalence of multimorbidity. Fifth, the reliance on aggregated population‑level data prevents individual‑level assessments of lipid‑lowering treatment utilization, adherence, and the distribution of ischemic stroke subtypes, which could refine etiologic and clinical insights. Finally, estimates for low‑SDI regions remain subject to greater statistical uncertainty due to chronic data sparsity, limited surveillance infrastructure, and fewer primary epidemiological studies in these settings.

## 5 Conclusion

In summary, the global epidemiology of HLIS demonstrates improving yet inequitably distributed trends. The reversal of the socioeconomic gradient, the key contributing role of demographic growth in low‐resource settings, and the slowing pace of risk reduction among younger birth cohorts collectively represent major population‐level public health observations. From a descriptive and modeling‐based inference perspective, addressing this burden calls for a dual approach: first, strengthening primary healthcare infrastructure, expanding access to affordable lipid management, and scaling stroke prevention services in low‐ and middle‐income countries to counteract rising absolute burden levels; and second, deploying demographically tailored interventions across all settings, with focused attention on middle‐aged men and younger adults. Shifting beyond sole reliance on economic indicators to account for context‐specific local factors associated with disease risk and resilience is critical to advancing equitable reductions in the global population burden of ischemic stroke.

## Author Contributions

**Xiaodong Yu**: writing – original draft, writing – review and editing, methodology, conceptualization, software. **Yi Zhang**: writing – original draft, writing – review and editing, data curation, investigation, validation. **Nannuan Liu**: formal analysis, visualization. **Yao Liu**: project administration, visualization, resources. **Genshan Gao**: writing – review and editing, project administration, formal analysis, supervision.

## Funding

The authors have nothing to report.

## Ethics Statement

All data were publicly available, no patient contact was made, and no individual identifiers were required. Therefore, ethical approval for the study was not required.

## Conflicts of Interest

The authors declare no conflicts of interest.

## Supporting information




**Supplementary Information**: brb371911‐sup‐0001‐SuppMat.docx

## Data Availability

The datasets used and/or analyzed during the current study are available from the corresponding author on reasonable request. To download the data used in these analyses, please visit the Global Health Data Exchange GBD 2021 website.
