# Supplementary material for: Lobal Burden of LDL‐C‐Related Ischemic Stroke: Unveiling Socioeconomic Reversals, Age‐Sex Disparities, and the Looming Threat of Slowing Cohort Improvements
Source: Brain Behav. 2026 Nov 13;16(11):e71911. doi: 10.1002/brb3.71911 (PMC13768746; doi:10.1002/brb3.71911)
Supplement: Supplementary file 1 [file BRB3-16-e71911-s001.docx]

Figure S1: Joinpoint regression analysis of age-standardized mortality rate (ASMR) for ischemic stroke attributable to high LDL-C

Figure S2: Joinpoint regression analysis of age-standardized DALY rate (ASDR) for ischemic stroke attributable to high LDL-C

Figure S3: Results of age-period-cohort model analysis for HLDL-Stroke mortality in global, 1990-2021. (A) The local drift with net drift values of ASMR for both sex; (B) Longitudinal age curves of ASMR; (C) Period effects of ASMR; (D) Cohort effects of ASMR.

Figure S4: Results of age-period-cohort model analysis for HLDL-Stroke DALY in global, 1990-2021. (A) The local drift with net drift values of ASDR; (B) Longitudinal age curves of ASDR; (C) Period effects of ASDR; (D) Cohort effects of ASDR.

Table S1 Analysis of age and sex differences in mortality and DALY rates of ischemic stroke attributable to high LDL-C.

|  |  |  | **Numbers** | | | **Rate** | | |
| --- | --- | --- | --- | --- | --- | --- | --- | --- |
| **Measure** | **Sex** | **Age** | **Val** | **Upper** | **Lower** | **Val** | **Upper** | **Lower** |
| Deaths | Male | 25-29 years | 620.53 | 873.36 | 315.39 | 0.21 | 0.29 | 0.11 |
| Deaths | Male | 30-34 years | 1347.43 | 1899.49 | 680.45 | 0.44 | 0.62 | 0.22 |
| Deaths | Male | 35-39 years | 2355.33 | 3290.38 | 1188.16 | 0.83 | 1.16 | 0.42 |
| Deaths | Male | 40-44 years | 4278.50 | 5938.65 | 2107.11 | 1.70 | 2.36 | 0.84 |
| Deaths | Male | 45-49 years | 8825.87 | 12375.51 | 4190.73 | 3.71 | 5.20 | 1.76 |
| Deaths | Male | 50-54 years | 17304.37 | 25007.30 | 7702.83 | 7.80 | 11.27 | 3.47 |
| Deaths | Male | 55-59 years | 25802.24 | 38491.44 | 10756.20 | 13.25 | 19.77 | 5.52 |
| Deaths | Male | 60-64 years | 45152.45 | 70521.84 | 17582.34 | 29.03 | 45.34 | 11.30 |
| Deaths | Male | 65-69 years | 57155.58 | 94854.17 | 19059.11 | 43.35 | 71.95 | 14.46 |
| Deaths | Male | 70-74 years | 70714.84 | 123678.41 | 22266.88 | 73.36 | 128.31 | 23.10 |
| Deaths | Male | 75-79 years | 64255.47 | 116342.55 | 18618.61 | 107.47 | 194.60 | 31.14 |
| Deaths | Male | 80-84 years | 70542.38 | 128920.82 | 18364.27 | 192.47 | 351.74 | 50.10 |
| Deaths | Male | 85-89 years | 58742.88 | 108511.07 | 15176.12 | 340.49 | 628.95 | 87.96 |
| Deaths | Male | 90-94 years | 26991.47 | 50038.76 | 6882.73 | 463.09 | 858.52 | 118.09 |
| Deaths | Male | 95+ years | 7292.85 | 13446.62 | 1901.69 | 482.32 | 889.30 | 125.77 |
| Deaths | Female | 25-29 years | 514.73 | 753.92 | 256.72 | 0.18 | 0.26 | 0.09 |
| Deaths | Female | 30-34 years | 919.93 | 1322.73 | 453.80 | 0.31 | 0.44 | 0.15 |
| Deaths | Female | 35-39 years | 1564.78 | 2234.11 | 774.01 | 0.56 | 0.80 | 0.28 |
| Deaths | Female | 40-44 years | 2744.56 | 3991.49 | 1374.54 | 1.11 | 1.61 | 0.55 |
| Deaths | Female | 45-49 years | 5072.54 | 7326.28 | 2438.47 | 2.15 | 3.11 | 1.03 |
| Deaths | Female | 50-54 years | 9331.17 | 13519.71 | 4476.77 | 4.19 | 6.06 | 2.01 |
| Deaths | Female | 55-59 years | 15077.06 | 22856.27 | 6705.16 | 7.50 | 11.37 | 3.34 |
| Deaths | Female | 60-64 years | 26533.73 | 41248.99 | 10356.29 | 16.13 | 25.07 | 6.30 |
| Deaths | Female | 65-69 years | 38649.27 | 63785.36 | 13454.90 | 26.84 | 44.29 | 9.34 |
| Deaths | Female | 70-74 years | 59189.65 | 104526.00 | 18495.43 | 54.08 | 95.50 | 16.90 |
| Deaths | Female | 75-79 years | 63306.66 | 113855.91 | 18529.83 | 87.81 | 157.92 | 25.70 |
| Deaths | Female | 80-84 years | 90191.96 | 165875.71 | 23305.16 | 177.09 | 325.69 | 45.76 |
| Deaths | Female | 85-89 years | 81912.85 | 153799.49 | 21620.58 | 287.73 | 540.23 | 75.94 |
| Deaths | Female | 90-94 years | 54332.95 | 100119.16 | 14960.27 | 450.49 | 830.12 | 124.04 |
| Deaths | Female | 95+ years | 25467.55 | 46452.66 | 7045.41 | 646.67 | 1179.52 | 178.90 |
| DALYs | Male | 25-29 years | 75556.34 | 106616.82 | 37193.69 | 25.41 | 35.85 | 12.51 |
| DALYs | Male | 30-34 years | 130760.67 | 183658.33 | 65212.51 | 42.80 | 60.11 | 21.34 |
| DALYs | Male | 35-39 years | 192961.61 | 268867.41 | 94325.42 | 68.17 | 94.98 | 33.32 |
| DALYs | Male | 40-44 years | 291071.10 | 403370.55 | 141471.93 | 115.43 | 159.97 | 56.10 |
| DALYs | Male | 45-49 years | 504330.00 | 704548.34 | 238563.21 | 212.03 | 296.20 | 100.29 |
| DALYs | Male | 50-54 years | 839404.57 | 1199778.82 | 370752.27 | 378.14 | 540.49 | 167.02 |
| DALYs | Male | 55-59 years | 1089041.26 | 1614051.13 | 446949.70 | 559.27 | 828.89 | 229.53 |
| DALYs | Male | 60-64 years | 1531782.55 | 2373608.86 | 591802.59 | 984.84 | 1526.07 | 380.49 |
| DALYs | Male | 65-69 years | 1628942.60 | 2743341.30 | 551613.47 | 1235.61 | 2080.92 | 418.42 |
| DALYs | Male | 70-74 years | 1621163.98 | 2867232.68 | 506764.27 | 1681.86 | 2974.57 | 525.74 |
| DALYs | Male | 75-79 years | 1183280.07 | 2158732.09 | 347928.52 | 1979.17 | 3610.72 | 581.95 |
| DALYs | Male | 80-84 years | 996803.31 | 1806882.79 | 263318.96 | 2719.65 | 4929.85 | 718.43 |
| DALYs | Male | 85-89 years | 638796.54 | 1175738.55 | 167044.43 | 3702.60 | 6814.82 | 968.22 |
| DALYs | Male | 90-94 years | 251701.78 | 464796.72 | 64035.97 | 4318.47 | 7974.55 | 1098.67 |
| DALYs | Male | 95+ years | 64338.56 | 117954.97 | 16536.29 | 4255.08 | 7801.04 | 1093.64 |
| DALYs | Female | 25-29 years | 88338.90 | 128745.40 | 43264.25 | 30.36 | 44.24 | 14.87 |
| DALYs | Female | 30-34 years | 130894.44 | 191734.31 | 64443.20 | 43.79 | 64.14 | 21.56 |
| DALYs | Female | 35-39 years | 177914.62 | 253288.06 | 88988.24 | 64.04 | 91.18 | 32.03 |
| DALYs | Female | 40-44 years | 243159.89 | 347729.09 | 120495.97 | 98.01 | 140.16 | 48.57 |
| DALYs | Female | 45-49 years | 364959.36 | 520824.95 | 174900.97 | 154.88 | 221.02 | 74.22 |
| DALYs | Female | 50-54 years | 550505.36 | 787972.51 | 259711.99 | 246.93 | 353.44 | 116.49 |
| DALYs | Female | 55-59 years | 741082.17 | 1097204.96 | 316747.77 | 368.69 | 545.86 | 157.58 |
| DALYs | Female | 60-64 years | 1000319.29 | 1543946.74 | 392456.30 | 608.06 | 938.51 | 238.56 |
| DALYs | Female | 65-69 years | 1189115.81 | 1940356.05 | 417199.06 | 825.72 | 1347.39 | 289.70 |
| DALYs | Female | 70-74 years | 1398468.52 | 2431908.45 | 440304.33 | 1277.75 | 2221.98 | 402.30 |
| DALYs | Female | 75-79 years | 1169732.06 | 2122145.28 | 340387.56 | 1622.43 | 2943.43 | 472.12 |
| DALYs | Female | 80-84 years | 1256237.45 | 2317058.36 | 325122.84 | 2466.54 | 4549.39 | 638.36 |
| DALYs | Female | 85-89 years | 897337.67 | 1679024.55 | 231616.80 | 3151.97 | 5897.70 | 813.57 |
| DALYs | Female | 90-94 years | 508909.74 | 941058.12 | 137872.74 | 4219.51 | 7802.57 | 1143.14 |
| DALYs | Female | 95+ years | 220513.67 | 403606.59 | 60154.29 | 5599.26 | 10248.33 | 1527.43 |

Table S2: Decomposition analysis of ischemic stroke attributable to high LDL-C.

| **Deaths** |  |  |  |  |  |  |  |  |
| --- | --- | --- | --- | --- | --- | --- | --- | --- |
| **Location** | **Sex** | **OD** | **Aging** | **Population** | **EC** | **AP** | **PP** | **ECP** |
| Global | Both | 281688.28 | 271191.6 | 486506.5 | -476009.79 | 96.27 | 172.71 | -168.98 |
| High SDI | Both | -42370.39 | 82429.82 | 61145.85 | -185946.06 | -194.55 | -144.31 | 438.86 |
| Low SDI | Both | 20595.17 | -591.45 | 25378.86 | -4192.24 | -2.87 | 123.23 | -20.36 |
| High-middle SDI | Both | 53797.51 | 128944.1 | 136387.8 | -211534.37 | 239.68 | 253.52 | -393.2 |
| Middle SDI | Both | 172877.74 | 93578.81 | 138263.7 | -58964.79 | 54.13 | 79.98 | -34.11 |
| Low-middle SDI | Both | 76839.3 | 19947.45 | 72611.94 | -15720.09 | 25.96 | 94.5 | -20.46 |
| Global | Male | 175067.54 | 132607.8 | 221816.7 | -179356.92 | 75.75 | 126.7 | -102.45 |
| High SDI | Male | -13540.55 | 39249.45 | 27701.58 | -80491.59 | -289.87 | -204.58 | 594.45 |
| Low SDI | Male | 10776.69 | -831.73 | 12724.35 | -1115.93 | -7.72 | 118.07 | -10.36 |
| High-middle SDI | Male | 41349.27 | 60457.09 | 60798.73 | -79906.54 | 146.21 | 147.04 | -193.25 |
| Middle SDI | Male | 97389.08 | 47175.45 | 68706.19 | -18492.55 | 48.44 | 70.55 | -18.99 |
| Low-middle SDI | Male | 39112.58 | 7281.94 | 34368.3 | -2537.66 | 18.62 | 87.87 | -6.49 |
| Global | Female | 4071779.75 | 2270894 | 5301645 | -3500759.6 | 55.77 | 130.2 | -85.98 |
| High SDI | Female | -205399.48 | 599814.2 | 597944.7 | -1403158.35 | -292.02 | -291.11 | 683.14 |
| Low SDI | Female | 960788.95 | 130388.8 | 883248.3 | -52848.19 | 13.57 | 91.93 | -5.5 |
| High-middle SDI | Female | 288090.3 | -28651.6 | 342107.3 | -25365.4 | -9.95 | 118.75 | -8.8 |
| Middle SDI | Female | 825969.74 | 1032408 | 1405820 | -1612258.7 | 124.99 | 170.2 | -195.2 |
| Low-middle SDI | Female | 2202824 | 897232 | 1726379 | -420787.03 | 40.73 | 78.37 | -19.1 |
| **DALYs** |  |  |  |  |  |  |  |  |
| Global | Both | 6464934.31 | 4236377 | 10708599 | -8480041.09 | 65.53 | 165.64 | -131.17 |
| High SDI | Both | -652232.87 | 1103527 | 1155514 | -2911273.09 | -169.19 | -177.16 | 446.35 |
| Low SDI | Both | 1819553.02 | 343680.2 | 1822339 | -346466.37 | 18.89 | 100.15 | -19.04 |
| High-middle SDI | Both | 543300.73 | -32460.7 | 681905.5 | -106144 | -5.97 | 125.51 | -19.54 |
| Middle SDI | Both | 960875.92 | 1946450 | 2826372 | -3811945.76 | 202.57 | 294.15 | -396.72 |
| Low-middle SDI | Both | 3794868.98 | 1690074 | 3378348 | -1273552.92 | 44.54 | 89.02 | -33.56 |
| Global | Male | 4071779.75 | 2270894 | 5301645 | -3500759.6 | 55.77 | 130.2 | -85.98 |
| High SDI | Male | -205399.48 | 599814.2 | 597944.7 | -1403158.35 | -292.02 | -291.11 | 683.14 |
| Low SDI | Male | 960788.95 | 130388.8 | 883248.3 | -52848.19 | 13.57 | 91.93 | -5.5 |
| High-middle SDI | Male | 288090.3 | -28651.6 | 342107.3 | -25365.4 | -9.95 | 118.75 | -8.8 |
| Middle SDI | Male | 825969.74 | 1032408 | 1405820 | -1612258.7 | 124.99 | 170.2 | -195.2 |
| Low-middle SDI | Male | 2202824 | 897232 | 1726379 | -420787.03 | 40.73 | 78.37 | -19.1 |
| Global | Female | 2393154.56 | 1983012 | 5403138 | -4992995.02 | 82.86 | 225.77 | -208.64 |
| High SDI | Female | -446833.4 | 541022.9 | 559032.6 | -1546888.83 | -121.08 | -125.11 | 346.19 |
| Low SDI | Female | 858764.07 | 210678.7 | 940260.8 | -292175.34 | 24.53 | 109.49 | -34.02 |
| High-middle SDI | Female | 255210.44 | -4985.74 | 339713.2 | -79517.06 | -1.95 | 133.11 | -31.16 |
| Middle SDI | Female | 134906.19 | 946800.6 | 1419637 | -2231531.05 | 701.82 | 1052.31 | -1654.14 |
| Low-middle SDI | Female | 1592044.99 | 791600.1 | 1646114 | -845669.51 | 49.72 | 103.4 | -53.12 |

OD: Overall Difference; EC: Epidemiological Change; AP:	Aging Percentage; PP: Population Percentage;

ECP: Epidemiological Change Percentage.

Table S3: Age-period-cohort analysis results of ischemic stroke attributable to high LDL-C.

|  |  | **Deaths** | | | **DALY** | | |
| --- | --- | --- | --- | --- | --- | --- | --- |
| **Age** | **type** | **val** | **low** | **up** | **val** | **low** | **up** |
| 27.5 | Age effect | 0.42 | 0.37 | 0.48 | 49.07 | 46.94 | 51.29 |
| 32.5 | Age effect | 0.73 | 0.67 | 0.81 | 70.40 | 67.89 | 73.00 |
| 37.5 | Age effect | 1.30 | 1.21 | 1.40 | 104.17 | 101.07 | 107.35 |
| 42.5 | Age effect | 2.51 | 2.38 | 2.65 | 164.95 | 160.89 | 169.12 |
| 47.5 | Age effect | 5.08 | 4.89 | 5.29 | 279.41 | 273.70 | 285.24 |
| 52.5 | Age effect | 9.58 | 9.29 | 9.87 | 451.48 | 443.96 | 459.13 |
| 57.5 | Age effect | 15.05 | 14.68 | 15.43 | 621.40 | 612.22 | 630.72 |
| 62.5 | Age effect | 29.80 | 29.22 | 30.39 | 1000.82 | 988.00 | 1013.82 |
| 67.5 | Age effect | 41.32 | 40.58 | 42.08 | 1170.71 | 1156.00 | 1185.62 |
| 72.5 | Age effect | 69.54 | 68.38 | 70.71 | 1583.66 | 1564.06 | 1603.51 |
| 77.5 | Age effect | 97.34 | 95.73 | 98.98 | 1778.84 | 1755.64 | 1802.36 |
| 82.5 | Age effect | 157.51 | 154.56 | 160.51 | 2227.03 | 2192.82 | 2261.78 |
| 87.5 | Age effect | 237.78 | 233.03 | 242.63 | 2645.45 | 2598.20 | 2693.56 |
| 92.5 | Age effect | 309.94 | 302.65 | 317.39 | 2985.60 | 2915.12 | 3057.78 |
| 97.5 | Age effect | 362.85 | 351.08 | 375.00 | 3269.62 | 3149.54 | 3394.28 |
| 1897 | Cohort RR | 2.98 | 2.74 | 3.24 | 2.81 | 2.53 | 3.11 |
| 1902 | Cohort RR | 2.59 | 2.48 | 2.70 | 2.43 | 2.31 | 2.56 |
| 1907 | Cohort RR | 2.26 | 2.20 | 2.33 | 2.14 | 2.08 | 2.21 |
| 1912 | Cohort RR | 2.05 | 2.00 | 2.10 | 1.95 | 1.91 | 2.00 |
| 1917 | Cohort RR | 1.75 | 1.71 | 1.79 | 1.67 | 1.64 | 1.70 |
| 1922 | Cohort RR | 1.57 | 1.54 | 1.61 | 1.51 | 1.49 | 1.54 |
| 1927 | Cohort RR | 1.48 | 1.46 | 1.51 | 1.44 | 1.41 | 1.46 |
| 1932 | Cohort RR | 1.31 | 1.29 | 1.34 | 1.28 | 1.26 | 1.30 |
| 1937 | Cohort RR | 1.18 | 1.16 | 1.20 | 1.16 | 1.15 | 1.18 |
| 1942 | Cohort RR | 1.00 | 1.00 | 1.00 | 1.00 | 1.00 | 1.00 |
| 1947 | Cohort RR | 0.88 | 0.86 | 0.90 | 0.90 | 0.89 | 0.92 |
| 1952 | Cohort RR | 0.84 | 0.82 | 0.86 | 0.87 | 0.86 | 0.89 |
| 1957 | Cohort RR | 0.77 | 0.75 | 0.79 | 0.81 | 0.80 | 0.82 |
| 1962 | Cohort RR | 0.70 | 0.67 | 0.72 | 0.75 | 0.74 | 0.77 |
| 1967 | Cohort RR | 0.64 | 0.61 | 0.67 | 0.71 | 0.69 | 0.73 |
| 1972 | Cohort RR | 0.60 | 0.56 | 0.63 | 0.68 | 0.66 | 0.70 |
| 1977 | Cohort RR | 0.57 | 0.52 | 0.63 | 0.66 | 0.63 | 0.68 |
| 1982 | Cohort RR | 0.55 | 0.48 | 0.62 | 0.64 | 0.61 | 0.67 |
| 1987 | Cohort RR | 0.51 | 0.43 | 0.61 | 0.62 | 0.58 | 0.66 |
| 1992 | Cohort RR | 0.46 | 0.34 | 0.61 | 0.57 | 0.52 | 0.63 |
| 1994.5 | Period RR | 1.17 | 1.15 | 1.18 | 1.13 | 1.12 | 1.14 |
| 1999.5 | Period RR | 1.06 | 1.05 | 1.08 | 1.04 | 1.03 | 1.06 |
| 2004.5 | Period RR | 1.00 | 1.00 | 1.00 | 1.00 | 1.00 | 1.00 |
| 2009.5 | Period RR | 0.86 | 0.85 | 0.87 | 0.88 | 0.87 | 0.89 |
| 2014.5 | Period RR | 0.77 | 0.76 | 0.78 | 0.80 | 0.79 | 0.81 |
| 2019.5 | Period RR | 0.73 | 0.72 | 0.74 | 0.76 | 0.75 | 0.77 |
| 27.5 | Local Drifts | -1.25 | -2.14 | -0.35 | -0.79 | -1.07 | -0.50 |
| 32.5 | Local Drifts | -1.18 | -1.73 | -0.62 | -0.77 | -0.96 | -0.57 |
| 37.5 | Local Drifts | -1.35 | -1.74 | -0.95 | -0.94 | -1.09 | -0.79 |
| 42.5 | Local Drifts | -1.57 | -1.86 | -1.28 | -1.15 | -1.27 | -1.02 |
| 47.5 | Local Drifts | -1.63 | -1.84 | -1.42 | -1.21 | -1.31 | -1.11 |
| 52.5 | Local Drifts | -1.70 | -1.86 | -1.54 | -1.32 | -1.40 | -1.24 |
| 57.5 | Local Drifts | -1.95 | -2.08 | -1.83 | -1.60 | -1.67 | -1.53 |
| 62.5 | Local Drifts | -2.16 | -2.25 | -2.06 | -1.84 | -1.91 | -1.78 |
| 67.5 | Local Drifts | -2.37 | -2.45 | -2.28 | -2.09 | -2.14 | -2.03 |
| 72.5 | Local Drifts | -2.36 | -2.44 | -2.29 | -2.12 | -2.18 | -2.06 |
| 77.5 | Local Drifts | -2.15 | -2.22 | -2.07 | -1.95 | -2.02 | -1.89 |
| 82.5 | Local Drifts | -2.09 | -2.17 | -2.02 | -1.94 | -2.01 | -1.87 |
| 87.5 | Local Drifts | -2.15 | -2.23 | -2.07 | -2.03 | -2.12 | -1.93 |
| 92.5 | Local Drifts | -2.28 | -2.41 | -2.15 | -2.17 | -2.32 | -2.02 |
| 97.5 | Local Drifts | -2.51 | -2.76 | -2.27 | -2.44 | -2.74 | -2.13 |
| 1 | Net drift | -1.95 | -2.04 | -1.87 | -1.66 | -1.70 | -1.62 |

Table S4: Deaths from Ischemic Stroke Attributable to High LDL-C between 1990 and 2021 in 204 countries.

| Deaths | Numbers(95%UI) | ASMR(95%UI) | Numbers(95%UI) | ASMR(95%UI) | EAPC(95%CI) |
| --- | --- | --- | --- | --- | --- |
| People's Republic of China | 1145.84 [373.96-1973.86] | 18.23 [5.52-33.43] | 3000.53 [925.16-5274.57] | 15.93 [4.83-28.08] | -0.4 [-0.65 to -0.15] |
| Mongolia | 0.57 [0.19-1.03] | 6.47 [1.96-11.83] | 1.2 [0.43-2.04] | 6.76 [2.22-12.22] | 0.1 [-0.33 to 0.52] |
| Montenegro | 0.64 [0.19-1.16] | 11.53 [3.45-20.75] | 1.47 [0.42-2.67] | 17.82 [5.05-32.72] | 1.76 [1.51 to 2.01] |
| Russian Federation | 884.91 [293.56-1473.95] | 56.71 [18.37-95.35] | 751.26 [248.92-1248.66] | 30.96 [10.34-51.32] | -2.81 [-3.37 to -2.25] |
| Principality of Andorra | 0.04 [0.01-0.07] | 8.71 [2.42-16.4] | 0.07 [0.02-0.14] | 3.95 [1.01-7.35] | -2.32 [-2.57 to -2.08] |
| Republic of the Philippines | 28.92 [10.25-48.99] | 14.45 [4.52-25.72] | 83.92 [28.27-141.33] | 12.72 [4.16-22.24] | -0.21 [-0.32 to -0.09] |
| Kingdom of Tonga | 0.04 [0.01-0.06] | 8.19 [2.42-14.5] | 0.06 [0.02-0.11] | 7.99 [2.39-14.16] | 0.06 [-0.08 to 0.19] |
| Republic of Uzbekistan | 19.37 [6.94-31.74] | 17.78 [6.21-29.46] | 37.16 [12.23-61.48] | 17.43 [5.58-29.6] | -0.72 [-1.1 to -0.34] |
| Independent State of Samoa | 0.07 [0.02-0.13] | 11.35 [3.39-20.7] | 0.11 [0.04-0.2] | 9.44 [2.91-17.5] | -0.67 [-0.77 to -0.57] |
| Republic of the Union of Myanmar | 45.09 [15.41-75.23] | 24.89 [7.88-43.34] | 70.76 [21.62-124.49] | 17.94 [5.15-32.36] | -1.23 [-1.31 to -1.15] |
| Republic of Indonesia | 104.42 [33.59-188.82] | 14.18 [4.11-26.7] | 330.19 [104.94-576.82] | 19.43 [5.62-35.48] | 1.1 [0.95 to 1.24] |
| Taiwan (Province of China) | 20.36 [6.85-34.15] | 16.86 [5.42-29.03] | 19.01 [5.97-32.54] | 4.28 [1.36-7.33] | -4.41 [-4.67 to -4.15] |
| Kingdom of Thailand | 37.73 [12.73-61.77] | 13.39 [4.38-22.15] | 86.89 [28.51-153.84] | 8 [2.63-14.15] | -2.3 [-2.57 to -2.03] |
| Republic of Ecuador | 4.31 [1.41-7.29] | 9.36 [2.94-16.14] | 7.15 [2.2-13.14] | 4.87 [1.48-8.99] | -1.92 [-2.22 to -1.62] |
| Democratic Socialist Republic of Sri Lanka | 20.54 [6.31-34.78] | 27.8 [8.13-48] | 42.62 [12.88-74.76] | 19.31 [5.55-34.17] | -0.48 [-0.76 to -0.2] |
| Republic of Tajikistan | 5.48 [1.78-9.57] | 22.12 [6.99-39.09] | 7.5 [2.26-12.78] | 17.61 [4.94-30.96] | -1.02 [-1.42 to -0.63] |
| Republic of Poland | 142.85 [45.25-241.44] | 35.6 [11.18-60.46] | 93.93 [27.73-165.6] | 11.97 [3.6-20.95] | -3.95 [-4.08 to -3.81] |
| Republic of the Congo | 1.3 [0.37-2.25] | 17.6 [4.73-31.46] | 2.63 [0.8-4.7] | 14.78 [4.35-26.53] | -0.82 [-0.93 to -0.7] |
| Ukraine | 319.53 [104.76-533.73] | 49.11 [15.86-82.5] | 195.4 [65.36-339.62] | 24.5 [8.3-42.43] | -3.01 [-3.35 to -2.68] |
| Republic of Italy | 165.11 [50.24-286.33] | 18.94 [5.72-32.83] | 110.01 [31.93-199.14] | 5.16 [1.53-9.24] | -4.42 [-4.68 to -4.16] |
| Lebanese Republic | 3.9 [1.29-6.89] | 21.99 [6.9-39.67] | 5.5 [1.66-9.91] | 8.27 [2.51-14.78] | -3.32 [-3.59 to -3.05] |
| Solomon Islands | 0.11 [0.03-0.21] | 13.46 [3.94-25.7] | 0.3 [0.08-0.55] | 12.96 [3.53-25.01] | -0.15 [-0.29 to -0.02] |
| Republic of Malawi | 3.33 [1.01-5.69] | 12.38 [3.6-21.77] | 7.63 [2.33-14.02] | 14.41 [4.14-26.59] | 0.29 [0.06 to 0.51] |
| Romania | 102.79 [32.74-172.19] | 46.45 [14.31-79.77] | 110.01 [32.96-194.4] | 26.18 [7.96-45.88] | -2.33 [-2.58 to -2.07] |
| Kingdom of Belgium | 27.52 [8.66-47.52] | 17.4 [5.5-29.85] | 12.69 [3.66-22.66] | 4.09 [1.21-7.2] | -4.56 [-4.74 to -4.38] |
| Malaysia | 13.58 [4.67-22.01] | 16.8 [5.52-27.82] | 31.54 [10.77-51.19] | 13.46 [4.41-22.5] | -0.49 [-0.65 to -0.32] |
| Republic of Austria | 25.7 [7.99-44.03] | 20.54 [6.48-34.95] | 9.21 [2.68-16.07] | 3.9 [1.17-6.75] | -5.75 [-6.13 to -5.36] |
| Canada | 28.99 [8.68-50.83] | 9.23 [2.73-16.2] | 27.08 [7.34-48.28] | 3.12 [0.87-5.51] | -3.97 [-4.21 to -3.74] |
| Republic of Vanuatu | 0.07 [0.02-0.12] | 16.55 [4.98-31.42] | 0.17 [0.06-0.29] | 14.09 [4.46-25.92] | -0.68 [-0.74 to -0.62] |
| Bosnia and Herzegovina | 11.99 [4.03-19.83] | 36.69 [12.09-62.3] | 16.77 [5.61-29.09] | 25.94 [8.75-44.86] | -1.43 [-1.6 to -1.25] |
| Cook Islands | 0.01 [0-0.02] | 10.73 [3.18-19.22] | 0.02 [0-0.03] | 6.03 [1.77-10.97] | -1.97 [-2.13 to -1.8] |
| Republic of Namibia | 0.77 [0.23-1.37] | 17.38 [4.99-31.53] | 1.54 [0.47-2.93] | 15.82 [4.66-30.64] | -0.54 [-0.82 to -0.26] |
| Republic of Ghana | 11.53 [3.55-19.65] | 24.36 [7.34-42.38] | 34.42 [10.59-59.04] | 27.06 [7.82-47.8] | 0.61 [0.39 to 0.84] |
| Republic of San Marino | 0.06 [0.02-0.1] | 14.66 [4.21-25.75] | 0.05 [0.01-0.1] | 4.25 [1.2-8.37] | -3.46 [-3.85 to -3.08] |
| Republic of Korea | 54.26 [17.69-94.56] | 27.24 [8.15-48.53] | 56.47 [15.63-103.71] | 6.22 [1.72-11.43] | -5.5 [-5.79 to -5.22] |
| Republic of Estonia | 8.7 [2.84-14.49] | 43.72 [14.25-73.05] | 2.75 [0.87-4.74] | 8.53 [2.77-14.42] | -6.8 [-7.43 to -6.16] |
| Grand Duchy of Luxembourg | 1.44 [0.43-2.48] | 27.41 [8.28-47.03] | 0.56 [0.16-0.98] | 4.36 [1.29-7.62] | -5.74 [-5.88 to -5.6] |
| Democratic People's Republic of Korea | 25.47 [8.05-45.91] | 20.02 [6.21-37.55] | 57.71 [19.35-101.15] | 18.98 [6.17-34] | -0.24 [-0.43 to -0.04] |
| Kingdom of the Netherlands | 30.45 [9.41-52.14] | 14.8 [4.61-25.23] | 24.61 [7.4-43.59] | 5.97 [1.82-10.52] | -3.44 [-3.74 to -3.13] |
| Socialist Republic of Viet Nam | 73.32 [22.84-135.04] | 21.32 [6.38-39.74] | 186.64 [59.16-334.31] | 22.95 [6.89-41.81] | 0.52 [0.39 to 0.66] |
| Republic of Paraguay | 3.11 [0.92-5.38] | 15.92 [4.6-27.72] | 5.71 [1.78-10.17] | 10.93 [3.36-19.54] | -0.96 [-1.17 to -0.75] |
| Republic of Chile | 12.88 [3.98-22.25] | 14.86 [4.52-26.04] | 15.31 [4.58-27.18] | 5.77 [1.74-10.23] | -2.53 [-2.74 to -2.31] |
| Republic of Malta | 0.73 [0.23-1.22] | 18.68 [5.95-31.49] | 0.51 [0.15-0.9] | 4.34 [1.31-7.68] | -4.77 [-5.03 to -4.52] |
| Republic of Croatia | 19.96 [6.21-34.21] | 39.25 [12.07-67.85] | 13.65 [4.07-24.36] | 13.32 [4.05-23.63] | -3.67 [-3.82 to -3.53] |
| Commonwealth of Dominica | 0.08 [0.02-0.14] | 14.12 [4.34-25.46] | 0.09 [0.03-0.16] | 12.04 [3.48-22.14] | -0.56 [-0.64 to -0.48] |
| Republic of Serbia | 58.01 [19.74-98.2] | 72.2 [23.43-125] | 69.66 [20.43-123.38] | 39.72 [11.66-70.16] | -2.57 [-2.85 to -2.29] |
| Eastern Republic of Uruguay | 8.05 [2.51-13.99] | 20.99 [6.56-36.54] | 6.42 [1.89-11.39] | 9.42 [2.9-16.45] | -2.73 [-2.94 to -2.52] |
| Republic of Kiribati | 0.03 [0.01-0.06] | 12.41 [3.67-22.46] | 0.07 [0.02-0.12] | 13.34 [4.3-23.79] | 0.2 [0.15 to 0.25] |
| Dominican Republic | 3.08 [0.97-5.22] | 10.53 [3.23-18.31] | 8.85 [2.75-16.51] | 9.22 [2.85-17.31] | 0.06 [-0.21 to 0.32] |
| Japan | 220.66 [68.4-385.1] | 14.73 [4.4-25.92] | 219.74 [62.71-397.71] | 3.68 [1.09-6.51] | -4.72 [-4.9 to -4.54] |
| State of Libya | 2.16 [0.65-3.82] | 12.98 [3.77-23.6] | 6.97 [2.32-12.71] | 15.48 [4.92-28.75] | 1.11 [0.88 to 1.33] |
| Republic of Cyprus | 1.58 [0.45-2.85] | 34.79 [10.06-64.09] | 1.42 [0.42-2.56] | 9.22 [2.71-16.72] | -4.58 [-4.94 to -4.21] |
| Republic of Peru | 6.71 [2.16-11.61] | 6.31 [1.98-11.02] | 12.58 [4-23.16] | 3.78 [1.2-6.97] | -2.23 [-2.68 to -1.78] |
| Turkmenistan | 3.67 [1.3-6.08] | 21.62 [7.46-36.63] | 8.92 [2.95-15.71] | 25.13 [8.09-45.02] | 0.19 [-0.31 to 0.69] |
| French Republic | 120.79 [35.22-207.75] | 13.33 [3.95-22.78] | 80.79 [23.2-142.26] | 3.88 [1.16-6.71] | -3.9 [-4.06 to -3.74] |
| Republic of Albania | 2.49 [0.75-4.29] | 15.45 [4.48-26.9] | 4.93 [1.46-8.92] | 12.09 [3.53-22.05] | -0.35 [-0.65 to -0.04] |
| Kingdom of Cambodia | 5.96 [1.93-10.44] | 18.61 [5.71-33.83] | 15.1 [4.56-27.32] | 17.58 [5.26-32.48] | -0.3 [-0.43 to -0.17] |
| Federative Republic of Brazil | 166.83 [56.14-276.44] | 23.4 [7.53-39.55] | 206.65 [68.5-353.03] | 8.62 [2.84-14.8] | -3.08 [-3.21 to -2.95] |
| Kyrgyz Republic | 7.95 [2.62-13.53] | 29.9 [9.59-51.55] | 7.08 [2.33-11.71] | 16.4 [5.15-27.87] | -2.45 [-2.81 to -2.09] |
| Democratic Republic of Timor-Leste | 0.26 [0.08-0.46] | 14.15 [4.02-26.16] | 1.03 [0.31-1.9] | 15.06 [4.42-28.59] | 0.39 [0.2 to 0.58] |
| Republic of Guyana | 0.95 [0.32-1.6] | 29.02 [9.38-50.07] | 0.93 [0.3-1.61] | 18 [5.61-31.66] | -1.02 [-1.25 to -0.79] |
| Kingdom of Spain | 102.93 [30.65-179.05] | 19.56 [5.76-34.15] | 51.09 [14.49-93] | 3.66 [1.07-6.58] | -5.48 [-5.74 to -5.23] |
| Republic of Colombia | 15.26 [4.99-25.55] | 10.35 [3.27-17.68] | 22.41 [6.93-39.54] | 3.99 [1.23-7.03] | -3.57 [-3.85 to -3.29] |
| Republic of Mozambique | 7.23 [2.33-12.3] | 16.09 [4.87-28.1] | 17.3 [5.5-29.46] | 20.52 [6.17-36.08] | 1.24 [1.06 to 1.41] |
| Federated States of Micronesia | 0.07 [0.02-0.12] | 17.02 [5.27-30.5] | 0.07 [0.02-0.13] | 14.26 [4.34-26.16] | -0.69 [-0.76 to -0.62] |
| Democratic Republic of the Congo | 12.86 [3.74-23.3] | 12.61 [3.35-23.74] | 27.14 [7.93-54.27] | 11.51 [3.01-23.7] | -0.42 [-0.48 to -0.36] |
| Republic of Azerbaijan | 5.2 [1.71-9.05] | 12.2 [3.96-21.89] | 7.92 [2.39-14.53] | 10 [2.84-18.56] | -0.3 [-0.57 to -0.03] |
| Islamic Republic of Afghanistan | 17.95 [5.51-32.57] | 31.06 [9.49-58.18] | 22.77 [7.76-40.29] | 29.16 [9.38-53] | -0.31 [-0.46 to -0.15] |
| Grenada | 0.18 [0.05-0.32] | 22.8 [6.9-39.04] | 0.12 [0.04-0.2] | 12.82 [3.8-22.84] | -1.78 [-1.99 to -1.58] |
| Hellenic Republic | 38.33 [11.1-67.16] | 27.24 [7.79-47.87] | 27.49 [7.48-50.26] | 7.64 [2.15-13.8] | -4.87 [-5.26 to -4.48] |
| People's Democratic Republic of Algeria | 19.83 [6.48-35.93] | 26.17 [7.78-48.48] | 48.92 [15.12-87.64] | 19.39 [5.54-35] | -0.75 [-0.81 to -0.68] |
| Portuguese Republic | 57.61 [17.59-99.23] | 47.27 [14.07-81.87] | 26.36 [7.55-46.87] | 7.89 [2.32-13.91] | -6.38 [-6.63 to -6.12] |
| Puerto Rico | 2.55 [0.79-4.45] | 7.9 [2.43-13.86] | 2.33 [0.67-4.19] | 2.5 [0.78-4.42] | -4.1 [-4.35 to -3.86] |
| Republic of Burundi | 3.01 [0.88-5.82] | 16.07 [4.36-31.44] | 3.22 [1.03-6.23] | 9.64 [2.81-19.08] | -2.27 [-2.52 to -2.03] |
| Gabonese Republic | 0.61 [0.17-1.06] | 13.1 [3.47-23.5] | 0.91 [0.26-1.6] | 12.29 [3.42-22.53] | -0.35 [-0.52 to -0.19] |
| Swiss Confederation | 15.38 [4.57-26.73] | 13.1 [3.98-22.67] | 8.42 [2.43-15.01] | 3.34 [0.99-5.89] | -4.3 [-4.45 to -4.16] |
| Georgia | 9.97 [3.02-17.61] | 17.72 [5.32-31.51] | 14.56 [4.34-26.09] | 22.21 [6.78-39.66] | 0.8 [0.12 to 1.48] |
| State of Kuwait | 0.49 [0.17-0.77] | 10.57 [3.39-17.67] | 1.76 [0.62-2.91] | 7.49 [2.34-13.06] | -0.86 [-1.99 to 0.28] |
| People's Republic of Bangladesh | 54.71 [16.62-104.65] | 14.13 [4.23-26.94] | 144.13 [39.13-275.88] | 12.78 [3.35-24.84] | -0.44 [-0.74 to -0.13] |
| Republic of Rwanda | 2.63 [0.77-5] | 12.82 [3.67-24.97] | 2.98 [0.83-5.95] | 6.98 [1.82-14.06] | -2.85 [-3.27 to -2.43] |
| Bolivarian Republic of Venezuela | 6.19 [2-10.83] | 7.67 [2.39-13.64] | 16.99 [5.03-30.31] | 6.32 [1.84-11.37] | -0.85 [-1.17 to -0.53] |
| Palestine | 2.72 [0.87-4.85] | 38.92 [12.05-70.88] | 4.2 [1.36-7.23] | 23.96 [7.21-42.59] | -1.63 [-1.91 to -1.35] |
| Republic of Fiji | 0.3 [0.1-0.52] | 12.38 [3.79-22.58] | 0.59 [0.21-1.04] | 11.55 [3.65-20.87] | -0.65 [-0.89 to -0.41] |
| Republic of Equatorial Guinea | 0.2 [0.06-0.38] | 14.75 [3.77-28.48] | 0.43 [0.13-0.8] | 12.4 [3.41-23.46] | -0.79 [-1.01 to -0.57] |
| Kingdom of Morocco | 32.9 [10.7-58.42] | 26.33 [8.08-47.87] | 76.39 [23.38-135.85] | 26.03 [7.66-46.54] | 0.09 [0.04 to 0.15] |
| Republic of Armenia | 3.74 [1.19-6.57] | 16.11 [4.98-28.77] | 4.89 [1.42-8.69] | 11.3 [3.29-20.09] | -2.1 [-2.47 to -1.73] |
| Commonwealth of the Bahamas | 0.14 [0.05-0.24] | 10.57 [3.35-18.24] | 0.25 [0.08-0.45] | 7.33 [2.29-13.29] | -1.21 [-1.37 to -1.05] |
| Republic of Bulgaria | 45.54 [15.27-76.62] | 50.02 [15.95-86.37] | 61.09 [18.72-104.27] | 41.87 [13.02-71.01] | -0.35 [-0.53 to -0.17] |
| Jamaica | 2.92 [0.88-5.18] | 15.57 [4.73-27.54] | 3.75 [1.07-6.72] | 11.07 [3.2-19.78] | -0.98 [-1.39 to -0.57] |
| Kingdom of Eswatini | 0.24 [0.06-0.44] | 12.19 [3.02-23.1] | 0.52 [0.14-0.97] | 13.68 [3.5-25.53] | 0.88 [0.47 to 1.3] |
| Guam | 0.05 [0.02-0.09] | 10.72 [3.42-18.83] | 0.07 [0.03-0.13] | 3.45 [1.21-5.95] | -3.25 [-3.69 to -2.8] |
| Republic of Belarus | 37.38 [12.27-63.14] | 29.79 [9.69-50.53] | 33.42 [10.23-58.17] | 20.2 [6.19-35.12] | -1.98 [-2.4 to -1.56] |
| Republic of India | 292.97 [95.18-521.45] | 8.31 [2.51-14.94] | 742.49 [251.81-1315.59] | 7.34 [2.39-13.13] | -0.52 [-0.66 to -0.39] |
| Republic of Singapore | 3.04 [1.01-5.01] | 16.97 [5.44-28.65] | 1.58 [0.47-2.77] | 1.92 [0.56-3.39] | -6.88 [-7.35 to -6.41] |
| Central African Republic | 1.18 [0.32-2.1] | 16.05 [4.12-29.32] | 1.89 [0.53-3.51] | 14.44 [3.77-28.43] | -0.42 [-0.5 to -0.35] |
| Republic of Yemen | 11.57 [3.66-21.3] | 30.53 [9.43-57.49] | 32.31 [10.58-59.63] | 29 [9.06-55.63] | -0.37 [-0.45 to -0.28] |
| Republic of Zimbabwe | 2.92 [0.87-5.28] | 10.44 [2.94-19.77] | 6.96 [2.17-12.85] | 14.64 [4.31-27.68] | 1.72 [1.16 to 2.28] |
| Republic of Suriname | 0.35 [0.11-0.58] | 15.38 [4.83-26.28] | 0.68 [0.21-1.17] | 11.73 [3.56-20.23] | -0.83 [-1.11 to -0.55] |
| Republic of El Salvador | 1.92 [0.61-3.28] | 6.86 [2.13-11.75] | 3.02 [0.9-5.34] | 4.46 [1.34-7.9] | -1.58 [-1.9 to -1.26] |
| United States Virgin Islands | 0.07 [0.02-0.12] | 10.97 [3.36-19.74] | 0.09 [0.03-0.15] | 4.98 [1.51-8.97] | -2.33 [-2.49 to -2.18] |
| Republic of Zambia | 1.85 [0.52-3.46] | 9.33 [2.53-17.95] | 5.14 [1.41-10.1] | 11.01 [2.94-21.64] | 0.42 [0.31 to 0.53] |
| Republic of Liberia | 1.42 [0.41-2.54] | 15.3 [4.38-28.04] | 2.51 [0.84-4.63] | 15.91 [5.05-29.57] | 0.15 [0.06 to 0.24] |
| Republic of Madagascar | 5.58 [1.69-10.29] | 14.28 [4.35-26.62] | 9.84 [3.12-17.63] | 13.44 [3.97-25.44] | -0.35 [-0.43 to -0.28] |
| Brunei Darussalam | 0.15 [0.05-0.26] | 20.7 [6.25-35.67] | 0.21 [0.07-0.35] | 10.44 [3.03-18.38] | -1.86 [-2.17 to -1.55] |
| United Mexican States | 35.78 [11.7-60.92] | 10.83 [3.38-18.81] | 55.43 [16.99-94.26] | 4.89 [1.46-8.43] | -2.6 [-2.75 to -2.46] |
| State of Qatar | 0.11 [0.04-0.19] | 21.41 [5.89-39.64] | 0.35 [0.11-0.62] | 8.71 [2.44-16.26] | -3.53 [-4.32 to -2.72] |
| Principality of Monaco | 0.2 [0.06-0.36] | 23.6 [6.92-42.43] | 0.12 [0.03-0.21] | 9.07 [2.73-15.83] | -3.23 [-3.43 to -3.02] |
| Republic of Seychelles | 0.1 [0.03-0.17] | 18.33 [6.11-30.56] | 0.12 [0.04-0.21] | 12.7 [4.06-21.78] | -0.85 [-1.06 to -0.64] |
| Republic of Slovenia | 6.13 [1.96-10.36] | 25.2 [8.05-42.57] | 3.93 [1.12-7] | 7.03 [2.04-12.36] | -4.12 [-4.35 to -3.89] |
| Republic of Botswana | 0.56 [0.16-1.04] | 14.86 [4-29.35] | 1.07 [0.32-1.89] | 10.4 [2.91-18.56] | -1.06 [-1.31 to -0.81] |
| Islamic Republic of Iran | 53.23 [19.02-86.81] | 27.81 [9.28-47.73] | 99.41 [32.3-167.1] | 14.75 [4.64-25.23] | -2.22 [-2.36 to -2.08] |
| Republic of Finland | 13.43 [4.23-23.01] | 18.55 [5.89-31.72] | 8.77 [2.57-15.57] | 5.27 [1.58-9.27] | -4.06 [-4.23 to -3.9] |
| Lao People's Democratic Republic | 4.51 [1.43-7.75] | 28.46 [8.62-51.06] | 6.65 [2.07-11.68] | 19.12 [5.47-34.21] | -1.46 [-1.58 to -1.35] |
| Federal Republic of Germany | 287.09 [89.41-493.18] | 21.26 [6.74-36.46] | 128.56 [38.26-226.72] | 5.24 [1.67-9.1] | -4.61 [-4.9 to -4.32] |
| State of Eritrea | 0.79 [0.23-1.54] | 11.84 [3.3-23.06] | 1.79 [0.52-3.32] | 10.48 [2.96-19.85] | -0.52 [-0.58 to -0.47] |
| Slovak Republic | 17.15 [5.56-29.27] | 29.95 [9.71-51.28] | 13.71 [3.95-24.36] | 14.25 [4.12-25.22] | -2.49 [-2.58 to -2.41] |
| Republic of Tunisia | 7.98 [2.62-14.2] | 21.18 [6.87-38.04] | 19.25 [5.92-34.97] | 16.49 [4.89-30.3] | -1.01 [-1.14 to -0.88] |
| Republic of Guinea-Bissau | 0.68 [0.2-1.21] | 21.68 [6.18-39.66] | 1.17 [0.39-2.08] | 22.65 [7.11-40.83] | 0.37 [0.28 to 0.45] |
| Kingdom of Lesotho | 0.59 [0.15-1.12] | 8.71 [2.31-16.57] | 1.14 [0.33-2.14] | 14.44 [3.97-27.12] | 2.59 [2.07 to 3.1] |
| Arab Republic of Egypt | 83.76 [28.23-157.87] | 45.14 [13.51-86.74] | 165.64 [56.6-274.99] | 36.87 [11.82-64.42] | -0.16 [-0.35 to 0.02] |
| Federal Republic of Somalia | 1.46 [0.37-3.05] | 9.59 [2.48-19.97] | 2.88 [0.78-5.64] | 7.68 [1.97-15.58] | -0.75 [-0.8 to -0.7] |
| Republic of Benin | 2.92 [0.88-5.26] | 17.2 [5.02-31.26] | 6.18 [1.87-11.11] | 15.7 [4.56-28.43] | -0.24 [-0.33 to -0.14] |
| Republic of the Gambia | 0.48 [0.14-0.84] | 17.72 [4.97-31.45] | 1.61 [0.48-2.92] | 20.54 [5.88-37.97] | 0.46 [0.36 to 0.56] |
| Kingdom of Norway | 13.15 [3.98-22.87] | 16.76 [5.22-28.97] | 5.23 [1.54-9.26] | 4.12 [1.23-7.25] | -4.75 [-4.93 to -4.57] |
| Kingdom of Sweden | 25.2 [7.44-43.39] | 14.6 [4.44-24.97] | 12.84 [3.56-23.1] | 4.45 [1.26-7.93] | -3.97 [-4.21 to -3.73] |
| Republic of Kazakhstan | 33.71 [10.77-57.23] | 30.38 [9.45-52.31] | 35.18 [11.06-61.55] | 25.25 [7.66-45.76] | -1.15 [-1.65 to -0.65] |
| Tuvalu | 0.01 [0-0.01] | 17.16 [5.16-31.1] | 0.01 [0-0.02] | 13.94 [4.33-25.66] | -0.73 [-0.79 to -0.68] |
| Republic of Kenya | 3.81 [1.18-7.12] | 6.23 [1.75-11.88] | 11.06 [3.39-20.66] | 7.24 [2.13-13.98] | 0.71 [0.61 to 0.82] |
| Republic of Mauritius | 1.9 [0.66-3.04] | 30.97 [10.19-51.03] | 1.74 [0.58-2.94] | 10.35 [3.4-17.63] | -4.72 [-5.27 to -4.17] |
| Republic of Haiti | 6.2 [2.01-10.95] | 26.73 [8.14-49.09] | 10.44 [3.25-19.91] | 20.34 [5.99-39.86] | -0.77 [-0.83 to -0.71] |
| Czech Republic | 67.27 [21.11-112.45] | 50.17 [15.75-84.44] | 21.38 [6.6-37.17] | 9.05 [2.86-15.64] | -5.94 [-6.37 to -5.52] |
| Republic of Costa Rica | 1.1 [0.34-1.95] | 6.89 [2.13-12.27] | 2.44 [0.75-4.28] | 4.33 [1.34-7.59] | -2.03 [-2.43 to -1.63] |
| Kingdom of Denmark | 13.76 [4.29-23.48] | 15.44 [4.89-26.16] | 7.76 [2.22-13.49] | 5.47 [1.62-9.45] | -3.73 [-3.93 to -3.54] |
| Republic of Cabo Verde | 0.25 [0.08-0.44] | 10.62 [3.28-18.47] | 0.61 [0.18-1.02] | 14.6 [4.28-24.84] | 0.75 [0.4 to 1.1] |
| Sultanate of Oman | 1.3 [0.41-2.3] | 23.15 [6.91-42.58] | 2.29 [0.78-3.89] | 16.52 [4.89-29.52] | -0.52 [-0.82 to -0.22] |
| Antigua and Barbuda | 0.08 [0.02-0.15] | 13.95 [4.23-24.92] | 0.08 [0.02-0.14] | 9.03 [2.67-16.02] | -1.58 [-1.83 to -1.33] |
| Republic of Guinea | 4.08 [1.16-7.21] | 14.63 [4.12-26.44] | 8.04 [2.51-14.95] | 17.38 [5.26-32.29] | 0.87 [0.72 to 1.02] |
| Republic of the Marshall Islands | 0.02 [0.01-0.03] | 16.1 [4.88-29.37] | 0.03 [0.01-0.05] | 14.33 [4.21-26.43] | -0.48 [-0.55 to -0.4] |
| United States of America | 301.17 [93.32-512.58] | 8.89 [2.8-15.06] | 269.63 [75.81-485.17] | 4.11 [1.19-7.34] | -3.09 [-3.49 to -2.7] |
| Republic of South Africa | 17.28 [5.56-30.48] | 9.89 [2.94-17.81] | 47.25 [14.52-84.62] | 13.03 [3.94-23.67] | 0.98 [0.46 to 1.51] |
| Hungary | 58.88 [19.44-96.12] | 43.06 [14.14-70.91] | 28.91 [9.54-48.36] | 13.49 [4.58-22.28] | -4.1 [-4.31 to -3.9] |
| Saint Lucia | 0.18 [0.05-0.31] | 25.65 [7.43-45.71] | 0.25 [0.07-0.46] | 11.26 [3.24-20.36] | -3.48 [-3.92 to -3.05] |
| Democratic Republic of Sao Tome and Principe | 0.08 [0.02-0.14] | 13.56 [4-24.87] | 0.15 [0.05-0.25] | 16.71 [4.81-29.27] | 0.89 [0.77 to 1] |
| North Macedonia | 8.96 [2.93-15.16] | 57.04 [18.09-97.76] | 13.98 [4.26-24.35] | 59.37 [17.33-106.07] | -0.21 [-0.71 to 0.3] |
| Republic of Guatemala | 1.58 [0.51-2.75] | 6.79 [2.03-12.27] | 3.93 [1.2-7.03] | 4.32 [1.28-7.82] | -1.92 [-2.25 to -1.58] |
| American Samoa | 0.02 [0.01-0.03] | 11.29 [3.32-20.06] | 0.03 [0.01-0.06] | 8.66 [2.7-15.48] | -1.1 [-1.26 to -0.94] |
| Kingdom of Bahrain | 0.28 [0.1-0.45] | 28.88 [8.82-49.06] | 0.71 [0.25-1.17] | 16.14 [4.68-28.48] | -2.39 [-2.85 to -1.93] |
| Northern Mariana Islands | 0.01 [0-0.02] | 12.79 [3.74-22.53] | 0.03 [0.01-0.05] | 8.59 [2.66-15.26] | -1.77 [-2.05 to -1.49] |
| Saint Vincent and the Grenadines | 0.11 [0.03-0.19] | 16.85 [4.97-30.14] | 0.13 [0.04-0.24] | 10.72 [3.16-19.06] | -1.34 [-1.59 to -1.08] |
| Australia | 23.78 [7.37-41.1] | 13.18 [4.01-22.9] | 20.77 [5.91-37.25] | 3.69 [1.06-6.57] | -4.3 [-4.43 to -4.17] |
| Kingdom of Saudi Arabia | 10.65 [3.42-19] | 22.88 [6.72-42.11] | 25.94 [8.78-42.09] | 18.17 [5.24-31.67] | -0.94 [-1.12 to -0.77] |
| Republic of Côte d'Ivoire | 4.67 [1.51-7.87] | 16.51 [4.9-29.66] | 13.77 [4.4-24.66] | 16.55 [5.15-30.41] | -0.01 [-0.21 to 0.18] |
| Kingdom of Bhutan | 0.2 [0.06-0.38] | 11.75 [3.43-22.15] | 0.57 [0.17-1.02] | 10.54 [3.02-19.1] | -0.35 [-0.45 to -0.25] |
| Republic of Mali | 3.2 [0.95-6.01] | 11.24 [3.08-20.99] | 7.27 [2.11-14.2] | 11.21 [3.04-22.16] | 0.25 [0.11 to 0.39] |
| Islamic Republic of Mauritania | 1.62 [0.5-2.95] | 19.6 [5.89-35.99] | 2.85 [0.87-5.46] | 16.28 [4.79-31.59] | -0.75 [-0.93 to -0.56] |
| United Kingdom of Great Britain and Northern Ireland | 176.81 [54.48-301.87] | 18.28 [5.68-31.14] | 67.47 [19.1-119.66] | 4.16 [1.2-7.32] | -5.04 [-5.28 to -4.8] |
| Republic of Latvia | 16.67 [5.41-27.83] | 47.51 [15.4-79.27] | 12.53 [3.83-22.17] | 26.36 [8.26-46.22] | -2.42 [-2.7 to -2.14] |
| Republic of Sierra Leone | 3.06 [0.88-5.4] | 17.47 [4.78-31.11] | 5.31 [1.59-9.44] | 17.4 [4.98-32.35] | 0.27 [0.09 to 0.45] |
| Union of the Comoros | 0.21 [0.06-0.38] | 14.98 [4.4-26.67] | 0.42 [0.13-0.73] | 11.13 [3.4-19.81] | -1.26 [-1.46 to -1.07] |
| Republic of Iceland | 0.38 [0.12-0.65] | 12.14 [3.79-20.75] | 0.28 [0.08-0.49] | 3.84 [1.11-6.65] | -3.82 [-4.01 to -3.62] |
| Republic of Niue | 0 [0-0.01] | 13.3 [4.1-25.34] | 0 [0-0] | 10.83 [3.52-19.6] | -0.9 [-0.98 to -0.83] |
| United Republic of Tanzania | 6.01 [1.71-11.2] | 7.61 [2.15-14.7] | 23.89 [7.14-41.41] | 12.6 [3.55-22.49] | 1.47 [1.14 to 1.81] |
| Argentine Republic | 42.53 [13.76-74.27] | 14.73 [4.69-25.79] | 30.91 [9.52-54.53] | 5.25 [1.62-9.23] | -2.96 [-3.19 to -2.74] |
| Islamic Republic of Pakistan | 49.61 [15.98-93.28] | 10.65 [3.23-20.11] | 105.9 [36.79-193.24] | 11.45 [3.68-21.18] | -0.05 [-0.21 to 0.12] |
| New Zealand | 5.1 [1.63-8.78] | 13.53 [4.26-23.26] | 4.96 [1.44-8.75] | 5.14 [1.51-9.05] | -3.38 [-3.55 to -3.2] |
| Republic of the Niger | 2.08 [0.59-4.05] | 11.11 [3.01-22.41] | 6.48 [1.81-12.6] | 11.54 [3.05-22.39] | 0.31 [0.25 to 0.38] |
| Republic of Djibouti | 0.09 [0.03-0.17] | 10.45 [2.79-21.19] | 0.42 [0.13-0.81] | 10.24 [2.93-20.06] | -0.26 [-0.35 to -0.18] |
| Republic of Trinidad and Tobago | 1.55 [0.51-2.64] | 22.53 [7.22-39.33] | 2.1 [0.65-3.61] | 11.48 [3.48-19.78] | -2.42 [-2.65 to -2.18] |
| Syrian Arab Republic | 9.84 [3.25-16.63] | 22.36 [6.98-38.91] | 18.42 [6.06-33.37] | 18.37 [5.92-34.19] | -1.13 [-1.35 to -0.91] |
| Independent State of Papua New Guinea | 1.25 [0.39-2.29] | 11.19 [3.22-20.94] | 3.15 [0.93-5.9] | 9.9 [2.74-19.27] | -0.52 [-0.63 to -0.41] |
| Republic of Uganda | 3.95 [1.13-7.48] | 8.35 [2.34-15.99] | 6.9 [1.79-13.49] | 6.63 [1.7-13.16] | -1.39 [-1.68 to -1.1] |
| Plurinational State of Bolivia | 3.14 [0.91-5.56] | 12.32 [3.44-21.9] | 5.66 [1.69-10.34] | 7.71 [2.16-14.1] | -1.46 [-1.6 to -1.32] |
| Barbados | 0.56 [0.17-1] | 18.12 [5.52-32.14] | 0.58 [0.17-1.02] | 11.13 [3.31-19.63] | -1.76 [-1.98 to -1.54] |
| Federal Democratic Republic of Nepal | 7.89 [2.41-14.23] | 11.11 [3.27-20.44] | 16.69 [4.53-31.63] | 8.85 [2.33-16.93] | -0.77 [-0.93 to -0.61] |
| Republic of Maldives | 0.14 [0.05-0.22] | 22.71 [7.18-38.89] | 0.29 [0.09-0.5] | 10.97 [3.18-19.52] | -2.71 [-2.82 to -2.6] |
| Republic of Iraq | 25.56 [8.3-44.11] | 34.65 [10.87-60.74] | 60.77 [20.27-102.26] | 33.18 [10.13-57.16] | -0.74 [-0.95 to -0.53] |
| Republic of Cameroon | 2.79 [0.79-5.25] | 8.35 [2.31-15.78] | 9.37 [2.61-18.13] | 9.95 [2.67-19.47] | 0.68 [0.26 to 1.1] |
| Republic of Moldova | 7.15 [2.27-12.22] | 21.46 [6.55-37.72] | 8.2 [2.53-14.28] | 13.43 [4.16-23.39] | -1.25 [-1.71 to -0.79] |
| Burkina Faso | 2.2 [0.64-4.16] | 6.77 [1.92-13.09] | 4.8 [1.35-9.56] | 6.74 [1.84-13.44] | 0.21 [0.09 to 0.32] |
| State of Israel | 4.44 [1.4-7.82] | 10.09 [3.13-17.76] | 3.95 [1.12-7.07] | 2.72 [0.78-4.84] | -4.75 [-4.96 to -4.54] |
| Belize | 0.06 [0.02-0.11] | 6.94 [2.16-12.22] | 0.16 [0.05-0.28] | 6.34 [1.91-11.09] | -0.7 [-1.26 to -0.12] |
| Republic of Angola | 3.4 [0.99-6.23] | 13.35 [3.66-25.19] | 9.85 [2.99-17.74] | 13.1 [3.51-23.75] | -0.31 [-0.41 to -0.21] |
| Republic of Cuba | 10.26 [3.22-18.1] | 10.85 [3.36-19.17] | 18.19 [5.35-32] | 8.63 [2.61-15.06] | -0.74 [-0.86 to -0.61] |
| Hashemite Kingdom of Jordan | 3.26 [1.1-5.43] | 32.27 [10.06-55.74] | 8.52 [2.84-14.62] | 16.03 [4.95-28.48] | -2.77 [-3.14 to -2.41] |
| Republic of Chad | 3.15 [0.87-5.94] | 13.16 [3.65-24.9] | 7.14 [2.09-13.68] | 16.15 [4.68-31.11] | 0.68 [0.49 to 0.86] |
| Federal Republic of Nigeria | 59.79 [17.14-112.41] | 16.98 [4.78-31.88] | 90.31 [29.74-152.62] | 13.29 [4.13-22.49] | -0.92 [-1.05 to -0.79] |
| Federal Democratic Republic of Ethiopia | 7.87 [2.25-16.76] | 5.82 [1.57-11.74] | 14.87 [4.3-28.16] | 4.46 [1.18-8.55] | -1.14 [-1.24 to -1.03] |
| Republic of Turkey | 61.78 [20.45-106.12] | 22.03 [7.03-38.56] | 97.21 [28.05-178.07] | 11.89 [3.41-22.16] | -1.87 [-2.18 to -1.55] |
| Republic of Palau | 0.01 [0-0.02] | 16.64 [4.87-30.38] | 0.02 [0.01-0.04] | 14.6 [4.39-26.14] | -0.27 [-0.35 to -0.18] |
| Republic of Honduras | 1.75 [0.55-3.1] | 10.46 [3.1-18.65] | 7.07 [2.25-12.5] | 14.16 [4.28-25.46] | 1.14 [0.9 to 1.38] |
| Republic of Senegal | 4.48 [1.37-7.66] | 17.05 [4.93-29.96] | 10.13 [2.99-18.29] | 16.49 [4.8-30.19] | -0.18 [-0.24 to -0.12] |
| Republic of Panama | 1.26 [0.4-2.18] | 9.37 [2.94-16.37] | 2.77 [0.81-4.93] | 5.98 [1.75-10.66] | -1.59 [-1.78 to -1.39] |
| Ireland | 6.59 [2.04-11.6] | 17.11 [5.25-30.26] | 3.14 [0.89-5.81] | 3.59 [1.02-6.62] | -5.01 [-5.25 to -4.77] |
| Republic of Lithuania | 11.98 [4.01-19.17] | 26.71 [8.97-42.88] | 11.65 [3.55-19.89] | 17.15 [5.37-28.83] | -1.74 [-2.04 to -1.44] |
| United Arab Emirates | 0.82 [0.29-1.37] | 27.26 [8.22-47.46] | 2.56 [0.95-4.14] | 18.46 [5.13-31.72] | 0.56 [-0.08 to 1.2] |
| Tokelau | 0 [0-0] | 15.2 [4.5-28.1] | 0 [0-0] | 10.77 [3.36-19.72] | -1.24 [-1.29 to -1.18] |
| Bermuda | 0.07 [0.02-0.12] | 12.92 [4.01-22.62] | 0.08 [0.02-0.14] | 5.13 [1.53-9.04] | -3.14 [-3.34 to -2.94] |
| Republic of South Sudan | 1.93 [0.52-3.66] | 9.55 [2.47-18.24] | 2.16 [0.62-4.12] | 8.08 [2.16-15.42] | -0.77 [-0.96 to -0.59] |
| Saint Kitts and Nevis | 0.12 [0.04-0.21] | 33.52 [10.29-59.1] | 0.09 [0.03-0.16] | 18.29 [5.49-31.8] | -1.8 [-1.99 to -1.61] |
| Greenland | 0.05 [0.02-0.09] | 28.12 [8.67-49.44] | 0.04 [0.01-0.07] | 9.06 [2.65-16.53] | -3.89 [-4.08 to -3.69] |
| Republic of Nauru | 0.01 [0-0.01] | 23.38 [7.23-41.8] | 0.01 [0-0.02] | 21.78 [7.06-39.48] | -0.38 [-0.67 to -0.09] |
| Republic of Sudan | 18.4 [5.98-33.85] | 23.78 [7.36-44.77] | 31.99 [9.84-57.67] | 19.68 [5.76-36.25] | -0.74 [-0.78 to -0.7] |
| Republic of Nicaragua | 0.95 [0.3-1.65] | 7.4 [2.23-13.05] | 1.86 [0.59-3.38] | 4.5 [1.4-8.28] | -1.44 [-1.64 to -1.24] |
| Togolese Republic | 1.71 [0.56-2.93] | 18.26 [5.51-32.32] | 5.23 [1.61-9.12] | 18.95 [5.56-33.18] | 0.02 [-0.13 to 0.17] |

Table S5：DALYs from Ischemic Stroke Attributable to High LDL-C between 1990 and 2021 in 204 countries.

| DALYs | Numbers(95%UI) | ASMR(95%UI) | Numbers(95%UI) | ASMR(95%UI) | EAPC(95%CI) |
| --- | --- | --- | --- | --- | --- |
| People's Republic of China | 30186.8 [10650.97-49388.7] | 385.65 [129.83-647.51] | 68505.66 [23132.04-114181.67] | 335.59 [112.75-566.25] | -0.42 [-0.6 to -0.24] |
| Mongolia | 18.92 [7.23-31.26] | 177.97 [64.34-303.15] | 45.05 [18.2-69.26] | 188.57 [69.89-304.34] | 0.19 [-0.14 to 0.53] |
| Montenegro | 13.49 [4.75-22.35] | 226.32 [77.9-381.81] | 26.35 [8.23-45.8] | 290.88 [90.59-506.19] | 1.01 [0.94 to 1.09] |
| Russian Federation | 18292.31 [6759.12-28503.51] | 1063.17 [380.56-1680.3] | 14566.04 [5228.26-23173.48] | 609.7 [222.89-962.69] | -2.64 [-3.18 to -2.09] |
| Principality of Andorra | 0.83 [0.27-1.42] | 158.7 [50.24-274.66] | 1.27 [0.41-2.18] | 77.89 [25.95-132.06] | -2.2 [-2.4 to -2] |
| Republic of the Philippines | 768.66 [299.48-1223.68] | 285.92 [101.45-471.63] | 2332.92 [888.58-3675.51] | 292.57 [106.97-482.68] | 0.22 [0.12 to 0.31] |
| Kingdom of Tonga | 1.02 [0.39-1.63] | 195.38 [69.66-319.48] | 1.46 [0.52-2.39] | 185.8 [64.52-307.19] | -0.11 [-0.21 to -0.02] |
| Republic of Uzbekistan | 556.22 [217.53-842.1] | 473.01 [178.39-732.02] | 1095.87 [402.84-1710.8] | 419.99 [145.94-676.87] | -0.93 [-1.23 to -0.63] |
| Independent State of Samoa | 2.03 [0.78-3.36] | 255.95 [93.09-430.68] | 3.11 [1.13-5.27] | 224.82 [79.09-394.98] | -0.47 [-0.55 to -0.4] |
| Republic of the Union of Myanmar | 1196.05 [448.48-1907.21] | 542.07 [190.13-893.45] | 1704.79 [576.18-2892.24] | 371.56 [121.51-641.36] | -1.37 [-1.45 to -1.29] |
| Republic of Indonesia | 3138.87 [1161.28-5280.02] | 328.53 [111.41-576.55] | 8955.94 [3178.77-15205.81] | 404.9 [132.32-697.84] | 0.74 [0.62 to 0.86] |
| Taiwan (Province of China) | 543.13 [201.52-841.63] | 373.6 [131.57-601.2] | 558.21 [196.16-882.85] | 134.32 [48.66-210.87] | -3.25 [-3.45 to -3.05] |
| Kingdom of Thailand | 1063.05 [401.17-1638.07] | 310.96 [110.05-496.55] | 2186.18 [833.34-3581.84] | 204.96 [79.3-333.99] | -1.86 [-2.08 to -1.65] |
| Republic of Ecuador | 104.76 [39.64-167] | 197.16 [70.35-323.81] | 152.44 [53.92-259.67] | 96.14 [33.36-165.14] | -2.2 [-2.49 to -1.9] |
| Democratic Socialist Republic of Sri Lanka | 456.63 [162.65-737.72] | 491.06 [163.17-813.07] | 846.83 [298.1-1413.99] | 343.11 [118.4-577.79] | -0.72 [-0.93 to -0.5] |
| Republic of Tajikistan | 137.54 [50.72-223.67] | 504.27 [180.34-838.04] | 203.16 [68.34-329.05] | 373.46 [116.09-621.91] | -1.24 [-1.55 to -0.93] |
| Republic of Poland | 2725.52 [939.19-4378.47] | 643 [220.91-1036.83] | 1724.38 [580.19-2881.98] | 236.47 [84-389.21] | -3.6 [-3.72 to -3.48] |
| Republic of the Congo | 35.97 [11.12-59.41] | 376.53 [109.99-636.76] | 74 [25.32-126.15] | 308.66 [95.75-538.54] | -0.91 [-1.03 to -0.79] |
| Ukraine | 6459.19 [2322.88-10223.64] | 927.57 [330.13-1478.88] | 3985.28 [1541.99-6593.6] | 516.24 [204.5-844.35] | -2.58 [-2.88 to -2.28] |
| Republic of Italy | 2676.22 [891.01-4468.4] | 300.73 [101.76-499.54] | 1519.58 [465.15-2684.68] | 85.99 [28.77-146.16] | -4.29 [-4.59 to -3.99] |
| Lebanese Republic | 93.3 [34.41-159.18] | 451.4 [159.69-771.83] | 116.29 [41.72-186.88] | 186.15 [67.55-297.7] | -2.95 [-3.24 to -2.65] |
| Solomon Islands | 3.69 [1.33-6.47] | 304.83 [100.34-554.43] | 9.49 [3.42-15.95] | 296.18 [98.31-521.77] | -0.11 [-0.25 to 0.02] |
| Republic of Malawi | 91.75 [29.92-151.71] | 263.95 [82.55-449.13] | 201.68 [68.53-346.44] | 297.78 [96-530.08] | 0.17 [-0.05 to 0.4] |
| Romania | 2025.93 [700.12-3296.43] | 802.71 [268.53-1319.43] | 1868.96 [596.21-3166.65] | 475.11 [155.76-793.44] | -2.25 [-2.52 to -1.98] |
| Kingdom of Belgium | 451.49 [152.52-745.05] | 288.04 [99.06-470.11] | 218.34 [69.65-372.12] | 84.84 [28.96-141.53] | -3.93 [-4.15 to -3.7] |
| Malaysia | 365.37 [146.24-568.05] | 400.3 [148.25-637.99] | 855.25 [326.93-1311.07] | 313.39 [116.45-487.43] | -0.63 [-0.75 to -0.5] |
| Republic of Austria | 422.14 [141.32-694.97] | 343.17 [118.61-556.85] | 195.37 [62.65-324.21] | 99.4 [34.63-160.13] | -4.18 [-4.51 to -3.84] |
| Canada | 579.77 [195.71-950.14] | 180.5 [61.1-295.02] | 605.55 [198.8-1009.13] | 83.84 [29.32-137.45] | -2.82 [-3.02 to -2.61] |
| Republic of Vanuatu | 2.24 [0.83-3.74] | 379.75 [128.55-653.61] | 5.56 [2.19-8.92] | 335.7 [120.38-566.1] | -0.56 [-0.62 to -0.49] |
| Bosnia and Herzegovina | 281.17 [102.31-438.04] | 740.6 [257.28-1188.45] | 321.2 [113.03-531.5] | 512.82 [184.05-842.33] | -1.46 [-1.58 to -1.33] |
| Cook Islands | 0.28 [0.1-0.46] | 242.28 [82.1-398.77] | 0.4 [0.14-0.66] | 160.51 [57.93-264.91] | -1.39 [-1.54 to -1.25] |
| Republic of Namibia | 19.3 [6.29-32.88] | 344.35 [106.38-611.57] | 36.07 [11.74-64.42] | 303.65 [96.02-554.25] | -0.66 [-0.92 to -0.39] |
| Republic of Ghana | 334.01 [119.73-539.94] | 547 [179.88-912.89] | 969.49 [336.95-1611.55] | 591.44 [189.66-1004.62] | 0.52 [0.32 to 0.72] |
| Republic of San Marino | 0.9 [0.29-1.51] | 245.96 [80.35-408.48] | 0.77 [0.24-1.42] | 88.91 [29.82-156.83] | -2.97 [-3.24 to -2.69] |
| Republic of Korea | 1375.82 [494.95-2280.71] | 531.9 [173.67-911.14] | 1235.25 [412.29-2082.04] | 135.12 [46-227.49] | -5.11 [-5.36 to -4.85] |
| Republic of Estonia | 173.94 [62.71-276.83] | 851.79 [308.35-1356.21] | 52.47 [18.05-85.89] | 192.99 [70.29-303.4] | -6.15 [-6.68 to -5.6] |
| Grand Duchy of Luxembourg | 23.46 [7.6-38.81] | 432.6 [141.83-713.67] | 8.96 [2.96-15.12] | 78.25 [26.56-129.41] | -5.54 [-5.72 to -5.36] |
| Democratic People's Republic of Korea | 719.64 [252.66-1209.29] | 466.37 [158.81-814.3] | 1504.12 [547-2523.36] | 459.96 [164.35-779.05] | -0.11 [-0.28 to 0.06] |
| Kingdom of the Netherlands | 551.92 [183.69-899.29] | 270.88 [92.2-437.46] | 406.87 [132.94-683.35] | 109.69 [37.36-180.36] | -3.34 [-3.58 to -3.11] |
| Socialist Republic of Viet Nam | 1588.33 [542.45-2768.78] | 417.38 [139.14-736.69] | 4140.01 [1461-7129.26] | 446.83 [152.03-787.82] | 0.53 [0.39 to 0.67] |
| Republic of Paraguay | 59.57 [20.28-97.29] | 283.97 [93.22-472.26] | 109.51 [39.5-185.31] | 197.98 [69.48-340.74] | -1.09 [-1.24 to -0.94] |
| Republic of Chile | 272.06 [92.91-443.07] | 285.22 [94.89-471.89] | 305.64 [105.66-503.47] | 118.48 [41.46-194.01] | -2.5 [-2.65 to -2.34] |
| Republic of Malta | 13.4 [4.64-21.79] | 324.19 [111.69-530.81] | 8.23 [2.66-14.09] | 79.41 [27.43-131.24] | -4.7 [-4.88 to -4.53] |
| Republic of Croatia | 374.15 [127.4-609.79] | 679.75 [223.93-1125.75] | 229.31 [74.49-388.85] | 240.04 [81.36-399.1] | -3.51 [-3.63 to -3.39] |
| Commonwealth of Dominica | 1.43 [0.48-2.48] | 244.65 [81.69-424.06] | 1.67 [0.52-2.9] | 209.38 [64.85-368.59] | -0.53 [-0.63 to -0.42] |
| Republic of Serbia | 1109.83 [396.45-1809.34] | 1193.49 [418.3-1987.11] | 1158.93 [360.09-1964.88] | 675.98 [214.65-1134.42] | -2.4 [-2.66 to -2.15] |
| Eastern Republic of Uruguay | 155.42 [53.36-257.24] | 400.99 [139.89-659.56] | 109.02 [35.88-181.67] | 183.72 [63.63-297.86] | -2.72 [-2.89 to -2.56] |
| Republic of Kiribati | 1.18 [0.45-1.86] | 321.92 [112.99-526.56] | 2.33 [0.91-3.72] | 327.88 [118.87-549.27] | 0.02 [-0.03 to 0.06] |
| Dominican Republic | 71.65 [26.33-114.33] | 203.27 [70.53-333.44] | 194.85 [70.56-333.43] | 195.62 [69.42-336.68] | 0.2 [0.04 to 0.35] |
| Japan | 4324.78 [1472.09-7165.24] | 268.42 [91.23-447.36] | 3856.49 [1198.87-6688.63] | 97 [34.01-155.52] | -3.53 [-3.69 to -3.38] |
| State of Libya | 55.79 [19.77-90.99] | 288.79 [94.58-481.37] | 197.79 [74.78-333.25] | 354.85 [125.67-622.36] | 1.05 [0.86 to 1.25] |
| Republic of Cyprus | 25.47 [7.74-44.35] | 457.82 [137.02-830.95] | 21.64 [7.02-37.47] | 123.04 [39.75-212.94] | -4.57 [-4.84 to -4.3] |
| Republic of Peru | 150.46 [54.7-244.02] | 127.93 [44.02-211.34] | 277.92 [101.02-467.73] | 82.13 [29.5-138.85] | -1.92 [-2.32 to -1.52] |
| Turkmenistan | 105.42 [41.76-166.63] | 540.65 [203.52-872.18] | 255.86 [95.75-424.75] | 625.27 [222.44-1072.52] | 0.3 [-0.15 to 0.76] |
| French Republic | 1870.9 [611.19-3122.9] | 214.09 [72.15-352.4] | 1327.92 [420.96-2268.17] | 82.72 [28.78-135.74] | -2.99 [-3.12 to -2.85] |
| Republic of Albania | 54.04 [18.9-86.39] | 289.81 [95.4-477.43] | 88.61 [29.09-153.74] | 210.02 [70.16-360.96] | -0.7 [-0.95 to -0.44] |
| Kingdom of Cambodia | 153.02 [56.08-249.68] | 377.12 [127.14-639.61] | 355.99 [118.69-625.18] | 329.83 [105.52-583.8] | -0.58 [-0.69 to -0.47] |
| Federative Republic of Brazil | 3767.45 [1418.38-5868.77] | 451.11 [159.62-727.59] | 4238.35 [1569.23-6757.24] | 171.32 [62.47-275.12] | -3.13 [-3.3 to -2.97] |
| Kyrgyz Republic | 189.17 [69.55-305.98] | 649.36 [231.51-1064.05] | 207.04 [76.79-330.57] | 411.58 [144.38-668.51] | -2.07 [-2.43 to -1.71] |
| Democratic Republic of Timor-Leste | 7.38 [2.55-12.28] | 291.03 [91.94-513.36] | 24.61 [8.4-42.65] | 305.91 [100.78-537.26] | 0.33 [0.14 to 0.52] |
| Republic of Guyana | 22.28 [8.43-35.8] | 604.56 [216.74-995.72] | 20.75 [7.37-34.94] | 346.87 [118.05-586.55] | -1.26 [-1.47 to -1.06] |
| Kingdom of Spain | 1659.47 [549.29-2776.99] | 307.25 [102.86-512.08] | 878.81 [277.22-1499.78] | 79.63 [26.99-131.81] | -4.42 [-4.72 to -4.13] |
| Republic of Colombia | 359.7 [134.91-567.59] | 211.8 [73.52-343.65] | 473.36 [167.68-781.06] | 85.45 [30.29-140.87] | -3.43 [-3.76 to -3.1] |
| Republic of Mozambique | 190.1 [65.84-312.26] | 345.21 [114.8-578.79] | 474.86 [168.48-787.56] | 450.2 [148.32-762.8] | 1.31 [1.12 to 1.49] |
| Federated States of Micronesia | 1.84 [0.67-3.03] | 389.73 [135.78-663.87] | 2.32 [0.87-3.79] | 331.45 [116.24-563.69] | -0.62 [-0.68 to -0.56] |
| Democratic Republic of the Congo | 365.08 [118.03-634.1] | 266.43 [80.27-479.16] | 731.57 [234.61-1352.76] | 232.91 [71.62-451.27] | -0.55 [-0.61 to -0.5] |
| Republic of Azerbaijan | 144.46 [53.41-236.19] | 293.08 [104.94-486.07] | 220.5 [78.48-375.3] | 228.71 [75.76-396.72] | -0.74 [-0.96 to -0.52] |
| Islamic Republic of Afghanistan | 454.34 [145.77-796.74] | 681.08 [217.5-1207.88] | 652.61 [239.21-1116.09] | 637.15 [226.4-1127.34] | -0.35 [-0.51 to -0.18] |
| Grenada | 3.41 [1.12-5.64] | 461.21 [159.12-751.74] | 2.38 [0.8-4.02] | 229.68 [74.51-394.39] | -2.12 [-2.3 to -1.94] |
| Hellenic Republic | 600.2 [191.86-1031.99] | 406.51 [131.12-698.02] | 381.73 [115.57-667.95] | 128.36 [42.5-217.16] | -4.38 [-4.67 to -4.09] |
| People's Democratic Republic of Algeria | 498.27 [182.78-840.53] | 476.84 [161.14-831.04] | 1120.26 [414.65-1868.92] | 348.53 [118.61-603.95] | -0.99 [-1.03 to -0.96] |
| Portuguese Republic | 953.78 [308.83-1583.23] | 723.95 [233.61-1209.12] | 363.02 [114.45-624.61] | 125.69 [42.36-209.34] | -6.18 [-6.42 to -5.95] |
| Puerto Rico | 47.13 [16.26-79.83] | 136.32 [46.68-231.63] | 42.29 [14.33-71.46] | 56.98 [20.87-92.14] | -3.19 [-3.38 to -3] |
| Republic of Burundi | 78.43 [25.39-141.26] | 352.39 [109.65-643.77] | 90.68 [31.87-165.26] | 203.34 [66.71-376.97] | -2.36 [-2.6 to -2.12] |
| Gabonese Republic | 14.93 [4.64-25.62] | 281.2 [84.71-482.15] | 23.8 [7.84-39.58] | 256.22 [76.98-438.32] | -0.44 [-0.58 to -0.29] |
| Swiss Confederation | 240.37 [78.29-402.75] | 214.35 [72.02-353.32] | 138.39 [44.57-231.08] | 66.99 [23.02-108.9] | -3.72 [-3.9 to -3.54] |
| Georgia | 214.72 [74.1-365.29] | 358.03 [121.71-610.2] | 269.1 [88.82-455.08] | 442.42 [149.96-740.06] | 0.67 [0.2 to 1.14] |
| State of Kuwait | 17.47 [7.29-25.67] | 252.96 [92.12-397.61] | 60.92 [25.32-91.46] | 178.54 [65.18-286.83] | -0.87 [-1.77 to 0.04] |
| People's Republic of Bangladesh | 1258.67 [417.76-2307.1] | 281.76 [87.7-525.65] | 2993.92 [922.99-5699.59] | 233.03 [68.56-442.68] | -0.6 [-0.78 to -0.43] |
| Republic of Rwanda | 73.34 [23.01-133.96] | 279.03 [84.94-524.08] | 80.58 [23.84-151.14] | 143.63 [41.42-279.16] | -3 [-3.4 to -2.61] |
| Bolivarian Republic of Venezuela | 140.87 [52.07-230.74] | 150.69 [52.26-254.04] | 333.24 [110.94-564.21] | 117.39 [38.72-200.29] | -1.13 [-1.42 to -0.84] |
| Palestine | 57.48 [20.2-96.47] | 712.24 [241.5-1230.19] | 97.95 [36.5-156.32] | 431.55 [144.94-728.06] | -1.7 [-1.92 to -1.48] |
| Republic of Fiji | 10.44 [4.02-16.69] | 301.56 [106.25-505.63] | 18.33 [7.21-29.18] | 264.59 [95.95-441.78] | -0.71 [-0.86 to -0.56] |
| Republic of Equatorial Guinea | 5.57 [1.81-10.08] | 319.39 [94.92-593.22] | 11.75 [4.07-20.01] | 256.12 [78.09-463.36] | -0.94 [-1.15 to -0.72] |
| Kingdom of Morocco | 798.3 [302.95-1376.08] | 564.4 [202.83-970.34] | 1795.62 [610.6-3033.95] | 536.94 [176.72-921.81] | -0.07 [-0.12 to -0.01] |
| Republic of Armenia | 92.94 [33.09-153.46] | 354.45 [121.57-595.27] | 107.58 [34.66-180.63] | 251 [82.05-420.92] | -1.95 [-2.26 to -1.64] |
| Commonwealth of the Bahamas | 3.18 [1.15-5.2] | 211.5 [73.7-354.89] | 5.48 [1.95-9.35] | 143.4 [49.43-249.6] | -1.25 [-1.37 to -1.14] |
| Republic of Bulgaria | 967.5 [350.38-1558.08] | 880.5 [309.83-1450.85] | 1099.02 [372.95-1848.17] | 775.22 [272.99-1271.21] | -0.28 [-0.44 to -0.12] |
| Jamaica | 51.53 [16.86-87.84] | 280.92 [92.88-475.17] | 62.61 [19.93-108.01] | 196.1 [62.72-338.57] | -0.99 [-1.4 to -0.59] |
| Kingdom of Eswatini | 5.93 [1.8-10.41] | 240.86 [67.82-436.21] | 13.44 [4.08-24.64] | 275.97 [79.25-505.41] | 0.84 [0.42 to 1.27] |
| Guam | 1.66 [0.64-2.58] | 239.08 [83.75-398.71] | 2.9 [1.09-4.64] | 141.34 [54.37-224.2] | -1.54 [-1.79 to -1.29] |
| Republic of Belarus | 844.17 [302.32-1373.19] | 651.91 [233.4-1066.77] | 699.55 [228.76-1153.41] | 432.54 [143.65-709.73] | -2.09 [-2.55 to -1.63] |
| Republic of India | 8017.31 [2879.36-13561.68] | 182.5 [61.38-315.72] | 18181.53 [6699.84-30903.64] | 157.55 [56.61-266.86] | -0.65 [-0.75 to -0.55] |
| Republic of Singapore | 81.3 [30.14-126.31] | 388.7 [137.07-620.05] | 59.44 [21.35-93.72] | 70.03 [25.02-110.88] | -5.7 [-5.87 to -5.54] |
| Central African Republic | 33.54 [10.31-58.23] | 337.24 [95.7-588.71] | 55.32 [17.93-99.64] | 295.64 [85.39-545.38] | -0.53 [-0.61 to -0.45] |
| Republic of Yemen | 307.57 [106.93-546] | 647.32 [211.11-1175.83] | 842.43 [294.9-1455.95] | 601.3 [204.1-1069.11] | -0.43 [-0.51 to -0.34] |
| Republic of Zimbabwe | 74.44 [24.16-127.11] | 209.19 [64.57-374.02] | 190.02 [64.45-334.57] | 305.84 [96.79-555.91] | 1.71 [1.19 to 2.24] |
| Republic of Suriname | 7.38 [2.68-11.74] | 302.87 [105.68-489.41] | 14.43 [4.94-23.58] | 233.94 [78.24-386.97] | -0.87 [-1.14 to -0.6] |
| Republic of El Salvador | 44.01 [16.21-69.51] | 148.02 [52.73-237.26] | 60.19 [20.02-99.38] | 94.24 [31.66-154.76] | -1.66 [-1.99 to -1.34] |
| United States Virgin Islands | 1.47 [0.5-2.43] | 196.36 [64.28-335.84] | 1.64 [0.54-2.82] | 95.44 [33.02-161.83] | -2.18 [-2.31 to -2.04] |
| Republic of Zambia | 51.38 [16.28-91.58] | 199.88 [60.98-368.23] | 140.46 [45.44-263.47] | 227.57 [66.56-439.7] | 0.26 [0.16 to 0.36] |
| Republic of Liberia | 35.99 [11.28-63.24] | 326.14 [98.23-578.9] | 69.07 [24.94-120.35] | 333.68 [113.14-595.83] | 0.09 [0 to 0.17] |
| Republic of Madagascar | 153.04 [50.33-270.99] | 317.59 [101.69-574.94] | 305.99 [107.67-526.66] | 294.06 [96.58-529.63] | -0.38 [-0.45 to -0.32] |
| Brunei Darussalam | 4.22 [1.61-6.64] | 440.55 [152.79-716.41] | 6.31 [2.33-10.06] | 212.58 [71.25-355.14] | -2.26 [-2.48 to -2.05] |
| United Mexican States | 813.9 [309.54-1281.22] | 203.5 [71.42-332.31] | 1276.37 [473.5-2013.56] | 103.74 [37-166.91] | -2.29 [-2.43 to -2.15] |
| State of Qatar | 4.17 [1.67-6.36] | 404.08 [128.55-696.09] | 18.13 [7.72-28.13] | 167.61 [54.68-293.11] | -3.24 [-3.87 to -2.6] |
| Principality of Monaco | 3.04 [0.93-5.37] | 390.97 [125.44-670.74] | 1.75 [0.55-2.97] | 159.59 [55.74-261.32] | -3.03 [-3.2 to -2.86] |
| Republic of Seychelles | 2.36 [0.85-3.73] | 417.64 [149.95-660.8] | 3.17 [1.18-5.01] | 283.24 [102.22-455.84] | -1.02 [-1.17 to -0.87] |
| Republic of Slovenia | 116.35 [39.96-187.16] | 474.14 [163.25-762.87] | 63.96 [20.55-108.85] | 131.59 [45.24-220.71] | -4.21 [-4.44 to -3.98] |
| Republic of Botswana | 14.73 [4.68-26.52] | 301.82 [89.69-556.72] | 28.92 [9.75-49.11] | 222.1 [69.71-382.7] | -0.99 [-1.2 to -0.77] |
| Islamic Republic of Iran | 1459.68 [572.31-2234.82] | 582.86 [213.48-932.68] | 2382.66 [890.95-3729.91] | 309.04 [110.02-495.57] | -2.19 [-2.31 to -2.06] |
| Republic of Finland | 260.92 [89.87-423.14] | 362.39 [127.28-581.5] | 157.49 [50.09-268.38] | 114.17 [39.12-186.94] | -3.72 [-3.87 to -3.58] |
| Lao People's Democratic Republic | 120.95 [42.15-197.94] | 616.53 [202.67-1048.17] | 173.4 [62.77-286.74] | 398.86 [135.84-678.14] | -1.56 [-1.67 to -1.46] |
| Federal Republic of Germany | 5015.68 [1670.97-8178.48] | 381.86 [131.66-613.11] | 2584.13 [851.14-4274.65] | 126.66 [44.97-204.64] | -3.64 [-3.92 to -3.36] |
| State of Eritrea | 26.26 [8.31-48.31] | 264.13 [77.82-493.95] | 54.59 [18.38-96.97] | 222.82 [67.77-401.75] | -0.67 [-0.73 to -0.61] |
| Slovak Republic | 370.08 [129.65-593.19] | 625.56 [220.18-1010.58] | 293.04 [93.87-497.98] | 307.76 [100.29-519.83] | -2.37 [-2.48 to -2.27] |
| Republic of Tunisia | 185.75 [67.14-313.37] | 403.4 [141.17-699.67] | 413.27 [140.64-709.15] | 323.22 [108.46-558.05] | -0.89 [-1.01 to -0.78] |
| Republic of Guinea-Bissau | 19.07 [6.05-33.35] | 492.76 [151.67-870.87] | 34.81 [12.54-58.22] | 491.13 [162.18-853.66] | 0.17 [0.09 to 0.26] |
| Kingdom of Lesotho | 13.02 [3.64-24.86] | 169.39 [46.71-322.24] | 27.32 [8.33-50.16] | 285.1 [84.3-527.81] | 2.5 [2.04 to 2.96] |
| Arab Republic of Egypt | 2091.64 [787.95-3823.13] | 859.5 [294.7-1595.71] | 4367.55 [1680.79-7202.52] | 740.77 [254.54-1222.12] | -0.11 [-0.28 to 0.06] |
| Federal Republic of Somalia | 46.96 [14.13-88.58] | 214.58 [59.99-422.31] | 96.34 [30.34-171.02] | 172.41 [49.51-325.75] | -0.78 [-0.83 to -0.73] |
| Republic of Benin | 68.01 [22.41-119.13] | 356.71 [113.37-624.99] | 153.16 [51.51-265.66] | 317.56 [100.79-559.71] | -0.34 [-0.44 to -0.24] |
| Republic of the Gambia | 13.42 [4.42-23.01] | 392.85 [123.89-666.58] | 41.06 [14-71.45] | 432.6 [137.77-750.07] | 0.24 [0.12 to 0.37] |
| Kingdom of Norway | 225.05 [73.64-371.47] | 306.44 [104.83-496.55] | 100.09 [31.57-168.05] | 91.89 [30.99-150.22] | -4.15 [-4.24 to -4.05] |
| Kingdom of Sweden | 421.59 [138.95-694.51] | 259.94 [90.03-417.99] | 222.16 [71.21-381.38] | 93.27 [31.91-154.15] | -3.52 [-3.66 to -3.38] |
| Republic of Kazakhstan | 852 [307.74-1364.32] | 686.22 [236.2-1117.55] | 848.56 [305.8-1382.48] | 509.11 [170.97-860.23] | -1.61 [-2.15 to -1.06] |
| Tuvalu | 0.23 [0.08-0.38] | 379.25 [131.32-646.14] | 0.31 [0.11-0.52] | 314.1 [107.6-539.59] | -0.66 [-0.71 to -0.62] |
| Republic of Kenya | 107.32 [35.88-194.37] | 139.05 [44.99-256.1] | 313.61 [107.04-536.01] | 152.79 [48.84-274.76] | 0.47 [0.37 to 0.57] |
| Republic of Mauritius | 50.56 [19.71-77.7] | 703.77 [256.43-1098.92] | 43.21 [16.11-67.49] | 244.77 [91.29-384.07] | -4.47 [-4.99 to -3.95] |
| Republic of Haiti | 150.43 [51.92-257.15] | 513.64 [168.98-904.46] | 249.78 [83.69-457.4] | 383.06 [122.79-715.5] | -0.82 [-0.9 to -0.74] |
| Czech Republic | 1237.74 [413.67-1996.52] | 898.42 [302.33-1445.82] | 419.35 [140.2-685.67] | 191.74 [68.44-306.38] | -5.3 [-5.57 to -5.03] |
| Republic of Costa Rica | 23.15 [8.24-37.94] | 134.05 [46.1-224.51] | 46.78 [16.54-76.38] | 84.69 [30-138.38] | -1.94 [-2.27 to -1.61] |
| Kingdom of Denmark | 242.56 [81.85-398.13] | 285.8 [99.65-461.11] | 130.55 [42.28-218.53] | 102.6 [34.72-168.75] | -3.65 [-3.82 to -3.48] |
| Republic of Cabo Verde | 5.63 [1.9-9.55] | 246.42 [84.7-414.28] | 13.61 [4.71-22.3] | 306.1 [100.9-509.87] | 0.45 [0.19 to 0.71] |
| Sultanate of Oman | 36.93 [13.83-60.95] | 522.86 [174.5-900.61] | 74.4 [30.21-114.56] | 346.29 [116.15-575.04] | -0.88 [-1.07 to -0.69] |
| Antigua and Barbuda | 1.42 [0.46-2.42] | 252.72 [84.08-424.1] | 1.56 [0.52-2.58] | 158.51 [50.77-267.33] | -1.7 [-1.92 to -1.48] |
| Republic of Guinea | 101.22 [33.01-171.81] | 317.47 [100.28-542.2] | 204.99 [71.17-358.93] | 373.52 [124.65-666.82] | 0.81 [0.67 to 0.95] |
| Republic of the Marshall Islands | 0.54 [0.21-0.9] | 353.46 [121.46-607.86] | 1.05 [0.39-1.73] | 324.27 [108.09-560.67] | -0.36 [-0.43 to -0.29] |
| United States of America | 6174.9 [2180.67-10000.9] | 189.56 [68.43-303.9] | 5993.97 [2015.82-10072.87] | 105.08 [36.77-172.19] | -2.39 [-2.68 to -2.1] |
| Republic of South Africa | 483.1 [182.3-782.5] | 234.78 [83.7-395.77] | 1124.55 [394.71-1882.59] | 263.01 [86.39-448.37] | 0.45 [0.01 to 0.89] |
| Hungary | 1244.28 [453.71-1930.94] | 869.72 [319.27-1345.3] | 575.31 [205.31-915.66] | 295.14 [111.32-459.6] | -3.87 [-4.06 to -3.68] |
| Saint Lucia | 3.38 [1.09-5.7] | 426.6 [134.23-729.86] | 4.4 [1.39-7.54] | 189.07 [59.27-324.94] | -3.17 [-3.54 to -2.8] |
| Democratic Republic of Sao Tome and Principe | 1.89 [0.62-3.2] | 303.35 [98.18-522.99] | 4.06 [1.43-6.64] | 369.06 [120.72-620.52] | 0.73 [0.6 to 0.86] |
| North Macedonia | 179.36 [63.94-288.94] | 1048.53 [361.51-1724.56] | 254.03 [82.69-427.95] | 905.74 [284.45-1565.26] | -0.76 [-1.12 to -0.4] |
| Republic of Guatemala | 40.31 [14.72-66.32] | 128.81 [42.44-219.64] | 84.24 [29.67-141.08] | 80.76 [27.19-138.11] | -1.98 [-2.28 to -1.68] |
| American Samoa | 0.54 [0.21-0.84] | 256.92 [86.36-422.08] | 0.91 [0.35-1.48] | 203.34 [75.57-345.9] | -0.99 [-1.13 to -0.86] |
| Kingdom of Bahrain | 7.72 [3.03-11.59] | 517.88 [176.97-844.77] | 20.61 [8.34-31.56] | 277.59 [96.61-463.54] | -2.61 [-2.99 to -2.23] |
| Northern Mariana Islands | 0.47 [0.19-0.73] | 278.17 [91.54-470.35] | 0.9 [0.34-1.43] | 196.09 [69.17-331.74] | -1.47 [-1.68 to -1.27] |
| Saint Vincent and the Grenadines | 2.05 [0.65-3.46] | 297.68 [94.11-503] | 2.54 [0.82-4.28] | 188.68 [60.99-320.39] | -1.42 [-1.6 to -1.24] |
| Australia | 430.04 [143.24-709.82] | 226.09 [75.23-374.1] | 374.77 [120.46-634.98] | 77.86 [26.31-128.01] | -3.64 [-3.81 to -3.47] |
| Kingdom of Saudi Arabia | 280.03 [99.52-468.08] | 471.61 [154.28-827.06] | 900.35 [335.65-1400.4] | 391.59 [126.8-642.88] | -0.71 [-0.88 to -0.55] |
| Republic of Côte d'Ivoire | 143.84 [51.81-235.33] | 367.63 [121.2-613.79] | 399.73 [148.03-677.11] | 361.07 [120.29-639.37] | -0.15 [-0.34 to 0.05] |
| Kingdom of Bhutan | 5.39 [1.94-9.34] | 245.08 [78.36-440.24] | 12.25 [4.08-21.01] | 209.81 [68.43-363.23] | -0.53 [-0.62 to -0.45] |
| Republic of Mali | 88.9 [29.66-155.97] | 241.4 [75.19-440.12] | 197.72 [63.87-359.47] | 236.66 [72.93-443.87] | 0.13 [0 to 0.27] |
| Islamic Republic of Mauritania | 41.82 [14.21-72.76] | 432.8 [141.83-763.59] | 69.42 [23.43-124.41] | 336.39 [109.43-615.8] | -0.97 [-1.14 to -0.8] |
| United Kingdom of Great Britain and Northern Ireland | 2973.58 [974.21-4917.51] | 313.96 [105.93-512.49] | 1180.13 [370.36-2003.54] | 84.5 [28.59-139.57] | -4.53 [-4.72 to -4.34] |
| Republic of Latvia | 321.09 [112.97-513.77] | 898.27 [316.56-1437.87] | 208.72 [68.35-350.72] | 492.37 [169.57-809.11] | -2.46 [-2.72 to -2.2] |
| Republic of Sierra Leone | 77.88 [25.09-132.83] | 387.59 [120.44-670.94] | 143.01 [48.77-239.16] | 380.73 [120.97-654.51] | 0.23 [0.05 to 0.4] |
| Union of the Comoros | 5.85 [1.91-9.94] | 325.99 [101.97-564.18] | 10.78 [3.74-17.84] | 238.59 [79.02-398.91] | -1.29 [-1.47 to -1.11] |
| Republic of Iceland | 6.84 [2.33-11.04] | 229.68 [79.62-366.63] | 5.01 [1.62-8.39] | 79.55 [27.07-130.88] | -3.52 [-3.65 to -3.4] |
| Republic of Niue | 0.07 [0.02-0.12] | 303.76 [104.06-519.63] | 0.05 [0.02-0.09] | 252.03 [87.34-424.71] | -0.82 [-0.89 to -0.76] |
| United Republic of Tanzania | 170.24 [54.78-296.96] | 169.52 [52.12-303.54] | 615.06 [208.19-1016.82] | 263.86 [83.94-445.26] | 1.22 [0.94 to 1.51] |
| Argentine Republic | 872.3 [306.5-1416.84] | 281.34 [98.13-461.64] | 663.42 [225.24-1083.69] | 116.59 [40.45-188.8] | -2.63 [-2.82 to -2.45] |
| Islamic Republic of Pakistan | 1232.48 [442.58-2191.72] | 228.89 [79.55-412.95] | 2853.31 [1089.7-4873.97] | 246.03 [87.83-431.18] | -0.03 [-0.19 to 0.13] |
| New Zealand | 93.2 [32.33-151.5] | 239.31 [83.28-388.71] | 83.19 [27.13-139.3] | 94.45 [32.03-155.99] | -3.21 [-3.42 to -3.01] |
| Republic of the Niger | 61.03 [19.78-111.33] | 239.34 [74.57-442.26] | 174.47 [55.85-322.15] | 234.26 [71.14-438.09] | 0.08 [0.02 to 0.14] |
| Republic of Djibouti | 2.81 [0.93-5.19] | 231.58 [68.18-445.11] | 13.06 [4.6-23.71] | 225.39 [72.15-419.17] | -0.26 [-0.34 to -0.19] |
| Republic of Trinidad and Tobago | 33.11 [11.77-53.22] | 418.82 [145.42-684.89] | 41.43 [14.18-68.38] | 219.83 [75.31-362.78] | -2.44 [-2.67 to -2.22] |
| Syrian Arab Republic | 262.35 [98.42-415.7] | 489.21 [169.29-801.28] | 462.99 [165.78-800.64] | 373.78 [131.08-648.39] | -1.35 [-1.57 to -1.13] |
| Independent State of Papua New Guinea | 37.97 [13.59-64.97] | 237.86 [76.52-422.54] | 92.72 [32.87-163.09] | 206.51 [65.15-380.77] | -0.59 [-0.69 to -0.5] |
| Republic of Uganda | 107.47 [33.75-189.1] | 182.08 [54.62-337.28] | 200.6 [62.21-356.14] | 147.99 [42.95-270.48] | -1.26 [-1.53 to -0.99] |
| Plurinational State of Bolivia | 73.72 [24.72-124.45] | 243.28 [76.55-421.17] | 122.28 [42.72-209.14] | 144.5 [47.2-251.6] | -1.69 [-1.85 to -1.53] |
| Barbados | 9.26 [3-15.97] | 308.26 [102.65-520.96] | 9.89 [3.19-16.93] | 192.49 [63.48-326.63] | -1.68 [-1.87 to -1.48] |
| Federal Democratic Republic of Nepal | 203.06 [69.17-351.74] | 232.72 [74.47-415.71] | 384.37 [114.95-710.03] | 175.56 [50.38-324.67] | -0.97 [-1.11 to -0.83] |
| Republic of Maldives | 4.28 [1.68-6.51] | 508.89 [185.19-820.78] | 7.27 [2.79-11.63] | 219.87 [76.8-364.73] | -3.12 [-3.25 to -2.98] |
| Republic of Iraq | 629.82 [232.66-1019.75] | 783.63 [276.49-1293.77] | 1529.02 [588.78-2439.45] | 668.41 [233.81-1116.89] | -1.03 [-1.19 to -0.87] |
| Republic of Cameroon | 76.78 [23.54-141.55] | 181.63 [53.93-338.69] | 264.56 [80.46-493.36] | 215.42 [60.63-406.22] | 0.64 [0.21 to 1.07] |
| Republic of Moldova | 156.97 [54.46-257.96] | 398.18 [133.45-664.59] | 187.55 [64.32-309.62] | 311.92 [108.74-512.53] | -0.53 [-0.97 to -0.09] |
| Burkina Faso | 61.34 [19.74-112.72] | 150.33 [46.6-281.39] | 129.98 [41.31-244.16] | 145.3 [43.32-277.98] | 0.05 [-0.02 to 0.12] |
| State of Israel | 92.77 [31.1-151.23] | 196.66 [66.39-320.2] | 85.03 [28.21-141.86] | 66.27 [22.47-108.49] | -3.96 [-4.16 to -3.76] |
| Belize | 1.26 [0.44-2.08] | 134.47 [46.03-223.74] | 3.45 [1.23-5.58] | 120.81 [41.42-200.75] | -0.69 [-1.19 to -0.19] |
| Republic of Angola | 101.04 [32.48-178.11] | 293.17 [88.18-530.94] | 281.36 [97.49-478.04] | 274 [85.14-481.63] | -0.43 [-0.51 to -0.35] |
| Republic of Cuba | 207.04 [72.35-346.98] | 206.96 [71.73-347.75] | 333.99 [112.37-565.12] | 168.07 [57.5-282.48] | -0.6 [-0.71 to -0.49] |
| Hashemite Kingdom of Jordan | 87.42 [33.55-135.75] | 677.37 [240.76-1117.86] | 240.49 [91.69-381.69] | 339.33 [120.04-562.77] | -2.72 [-3.06 to -2.39] |
| Republic of Chad | 78.29 [24.42-141.07] | 287.32 [87.25-521.47] | 192.14 [62.27-344.95] | 346.34 [104.35-644.22] | 0.6 [0.41 to 0.78] |
| Federal Republic of Nigeria | 1451.76 [466.67-2580.64] | 350.14 [107.72-633.54] | 2375.57 [857.91-3790.16] | 276.21 [92.78-452.49] | -0.89 [-1.03 to -0.75] |
| Federal Democratic Republic of Ethiopia | 250.04 [77.46-502.21] | 135.89 [39.48-269.17] | 408.34 [134.09-732.95] | 98.61 [31.02-178.72] | -1.32 [-1.44 to -1.2] |
| Republic of Turkey | 1485.67 [561.94-2405.41] | 450.11 [159.51-751.21] | 2001.57 [665.18-3393.06] | 225.5 [74.08-387.71] | -2.33 [-2.52 to -2.13] |
| Republic of Palau | 0.37 [0.13-0.6] | 384.51 [128.29-658.24] | 0.7 [0.26-1.14] | 340.78 [119.67-561.15] | -0.3 [-0.36 to -0.24] |
| Republic of Honduras | 42.77 [15.43-71.01] | 212.29 [70.63-362.16] | 148.27 [51.22-249.99] | 255.16 [86.06-439.63] | 0.78 [0.61 to 0.95] |
| Republic of Senegal | 119.49 [39.88-197.31] | 379.86 [119.85-640.69] | 254.2 [82.97-428.88] | 343.58 [108.45-598.18] | -0.4 [-0.46 to -0.34] |
| Republic of Panama | 26.02 [9.36-43.08] | 178.95 [62.11-301.47] | 49.8 [16.86-85.16] | 110.7 [37.68-189.41] | -1.69 [-1.86 to -1.52] |
| Ireland | 116.03 [38.39-194.3] | 286.3 [95.51-479.03] | 52.32 [17.09-91.59] | 64 [21.49-110.9] | -5.01 [-5.25 to -4.77] |
| Republic of Lithuania | 262.57 [98.33-403.04] | 582.91 [219.46-896.52] | 209.11 [71.34-339.05] | 353.82 [127.1-559.27] | -1.58 [-1.88 to -1.29] |
| United Arab Emirates | 29.5 [12.17-44.93] | 603.24 [202.12-996.1] | 134.3 [59.72-198.2] | 363.68 [116.93-595.36] | -0.4 [-0.85 to 0.06] |
| Tokelau | 0.04 [0.01-0.07] | 326.45 [107.52-571.04] | 0.04 [0.01-0.06] | 246.34 [88.93-418.55] | -1.03 [-1.08 to -0.98] |
| Bermuda | 1.41 [0.49-2.35] | 235.73 [80.66-394.69] | 1.38 [0.44-2.34] | 97.51 [32.88-163.75] | -2.92 [-3.15 to -2.68] |
| Republic of South Sudan | 50.03 [14.85-91.6] | 207.78 [60.11-390.55] | 62.26 [20.55-110.46] | 177.01 [54.57-320.86] | -0.74 [-0.92 to -0.56] |
| Saint Kitts and Nevis | 2.22 [0.74-3.77] | 602.67 [203.83-1009.43] | 1.92 [0.66-3.18] | 317.97 [104.12-537.63] | -2 [-2.2 to -1.8] |
| Greenland | 1.41 [0.54-2.21] | 519.73 [174.43-863.87] | 1.03 [0.38-1.67] | 177.99 [60.32-297.72] | -3.69 [-3.84 to -3.54] |
| Republic of Nauru | 0.26 [0.1-0.41] | 560.56 [196.26-927.75] | 0.31 [0.12-0.49] | 525.74 [192.23-878.98] | -0.37 [-0.65 to -0.08] |
| Republic of Sudan | 474.64 [163.81-840.78] | 515.2 [175.99-929.8] | 870.42 [305.69-1525.49] | 429.71 [138.67-752.2] | -0.68 [-0.71 to -0.65] |
| Republic of Nicaragua | 22.56 [8.41-37.12] | 150.88 [52.56-254.46] | 44.17 [16.55-74.43] | 93.7 [33.33-160.27] | -1.5 [-1.64 to -1.36] |
| Togolese Republic | 48.12 [17.22-78.09] | 401.64 [134.39-677.18] | 149.25 [51.95-249.27] | 406.72 [129.23-700.87] | -0.06 [-0.23 to 0.1] |
